# Humoral Activity of Cord Blood-Derived Stem/Progenitor Cells: Implications for Stem Cell-Based Adjuvant Therapy of Neurodegenerative Disorders

**DOI:** 10.1371/journal.pone.0083833

**Published:** 2013-12-31

**Authors:** Edyta Paczkowska, Katarzyna Kaczyńska, Ewa Pius-Sadowska, Dorota Rogińska, Miłosz Kawa, Przemysław Ustianowski, Krzysztof Safranow, Zbigniew Celewicz, Bogusław Machaliński

**Affiliations:** 1 Department of General Pathology, Pomeranian Medical University, Szczecin, Poland; 2 Department of Obstetrics and Gynecology, Pomeranian Medical University, Szczecin, Poland; French Blood Institute, France

## Abstract

**Background:**

Stem/progenitor cells (SPCs) demonstrate neuro-regenerative potential that is dependent upon their humoral activity by producing various trophic factors regulating cell migration, growth, and differentiation. Herein, we compared the expression of neurotrophins (NTs) and their receptors in specific umbilical cord blood (UCB) SPC populations, including lineage-negative, CD34^+^, and CD133^+^ cells, with that in unsorted, nucleated cells (NCs).

**Methods and Results:**

The expression of NTs and their receptors was detected by QRT-PCR, western blotting, and immunofluorescent staining in UCB-derived SPC populations (i.e., NCs vs. lineage-negative, CD34^+^, and CD133^+^ cells). To better characterize, global gene expression profiles of SPCs were determined using genome-wide RNA microarray technology. Furthermore, the intracellular production of crucial neuro-regenerative NTs (i.e., BDNF and NT-3) was assessed in NCs and lineage-negative cells after incubation for 24, 48, and 72 h in both serum and serum-free conditions. We discovered significantly higher expression of NTs and NT receptors at both the mRNA and protein level in lineage-negative, CD34^+^, and CD133^+^ cells than in NCs. Global gene expression analysis revealed considerably higher expression of genes associated with the production and secretion of proteins, migration, proliferation, and differentiation in lineage-negative cells than in CD34^+^ or CD133^+^ cell populations. Notably, after short-term incubation under serum-free conditions, lineage-negative cells and NCs produced significantly higher amounts of BDNF and NT-3 than under steady-state conditions. Finally, conditioned medium (CM) from lineage-negative SPCs exerted a beneficial impact on neural cell survival and proliferation.

**Conclusions:**

Collectively, our findings demonstrate that UCB-derived SPCs highly express NTs and their relevant receptors under steady-state conditions, NT expression is greater under stress-related conditions and that CM from SPCs favorable influence neural cell proliferation and survival. Understanding the mechanisms governing the characterization and humoral activity of subsets of SPCs may yield new therapeutic strategies that might be more effective in treating neurodegenerative disorders.

## Introduction

Neurodegenerative diseases (NDs), such as amyotrophic lateral sclerosis, Alzheimer's disease, Huntington's disease, age-related macular degeneration, and Parkinson's disease are characterized clinically by their subtle onset but chronic progression and involve the degeneration of defined neuronal phenotypes in the central nervous system (CNS). Despite substantial research and the development of a number of neuroprotective drugs to treat NDs and to improve patient survival, no effective therapy for these diseases is currently available. Recently, stem cell-based therapy has been considered a novel therapeutic strategy for this group of disorders. Populations of stem cells from a variety of sources have been implicated in the regeneration of damaged neural cells. Human umbilical cord blood (UCB) is an attractive source of transplantable cells for use in regenerative medicine. As widely disseminated in the literature, human UCB is enriched in stem/progenitor cells (SPCs) that are able to give rise to multiple neural lineage cell types [1], [2]. In addition to findings from numerous in vitro experiments [3]–[6], several in vivo findings have provided data on the ameliorative effects of UCB-derived cells when transplanted into animal models of neurodegenerative diseases [7]–[9]. Therapeutic approaches involving the transplantation of stem cells focuses primarily on the replacement of lost neurons and the restoration of neural tissue structure. Although these experimental studies demonstrate that UCB-derived cells are capable of surviving transplantation, convincing evidence that they are able to differentiate into mature neurons is lacking. The reported beneficial effects of stem cell-based therapy may depend on the trophic activity of producing various cytokines, including neurotrophins (NTs), which regulate the growth, differentiation, and migration of neural SPCs. In recent years, numerous studies have shown that stem cell transplantation elicits neurogenesis and angiogenesis by releasing neuroprotective factors (e.g., brain-derived neurotrophic factor (BDNF) and nerve growth factor (NGF)) [10].

Despite the efforts made and the encouraging results reported, unresolved questions remain regarding the optimal population of stem cells that should be used to provide the best outcome in in vivo transplantation [11], [12]. The characterization of SPC subsets and an assessment of their ability to produce various NTs in vitro may stimulate the field of regenerative medicine by offering novel targets. In this context, identification of the optimal SPC population for neural tissue repair is paramount to the differentiation of transplanted stem cells. Insights into NT production by stem cells may help in devising more effective therapies and introduce widely extendable clinical applications.

Growing evidence suggests that UCB cell-induced neuroprotection involves anti-inflammatory and immunomodulatory effects, and that neurotrophic factors act through paracrine and/or autocrine interactions between transplanted UCB-derived cells and the neural microenvironment [13]–[15]. NTs regulate the growth, differentiation, and migration of neural cells and have been proposed to act as therapeutic agents for the treatment of neurodegenerative disorders [16]. However, NTs generally do not cross the blood-brain barrier to any substantial degree, and direct injection into neural tissue to target the effects of NTs is difficult. Three distinct families of neurotrophic factors have been characterized in recent years: classical NTs (e.g., NGF, BDNF, neurotrophin 3 (NT-3), and neurotrophin 4 (NT-4)), the glial cell line-derived neurotrophic factor (GDNF) family of ligands (GDNF, neurturin (NRTN), artemin (ARTN), and persephin (PSPN)), and neuropoietic cytokines [17]. The recently described proteins mesencephalic astrocyte-derived neurotrophic factor (MANF) and cerebral dopamine neurotrophic factor (CDNF) represent modern, evolutionary conserved neurotrophin family members that protect and functionally restore dopaminergic neurons [17]. Pigment epithelium-derived factor (PEDF) is a serine protease inhibitor (serpin) protein that exhibits well-documented neuroprotective and anti-angiogenic attributes [18]. The angiogenic-specific effect and neurotrophic activity of vascular endothelial growth factor (VEGF) also makes this trophic protein an attractive target for improving regenerative processes in a damaged CNS [17].

NTs activate two classes of receptors: neurotrophin receptor (p75NTR), which is a member of the tumor necrosis factor (TNF) family, and the more specific Trk receptors, which possess internal tyrosine kinase activity [19]. NTs share the same low-affinity p75NTR neurotrophin receptor but use different members of the Trk receptor tyrosine kinase family for high-affinity binding and signal transduction. NGF preferentially binds to TrkA, BDNF and NT-4 and -5 preferentially activate TrkB, and NT-3 acts via TrkC. GDNF signals through a novel class of glycosylphosphatidylinositol (GPI)-anchored proteins (i.e., GDNF family receptor alpha (GFRα) 1–4), and GDNF preferentially binds to GFRα1 [20].

Because the autocrine production of various soluble factors (including NTs) by SPCs can be increased during a stress response, we hypothesized that the short-term incubation of UCB-derived cells under non-physiological conditions can augment this phenomenon [21]. In this study, we studied the effect of stress-related serum-free conditions on the production of neuroprotective NTs using UCB-derived SPCs. In experimental studies, transplanted cells that are administered into vitreous or cerebrospinal fluid could occur in resemble conditions. Moreover, we determined whether lineage-negative-conditioned medium (CM) containing trophic factors, including NTs secreted by lineage-negative cells, could favor neural cell proliferation and survival.

Thus, the aim of our study was to determine whether different SPC populations are able to produce noticeable concentrations of neurotrophic factors because this has practical implications for stem cell-based therapies. Identifying the specific SPC-enriched population to effectively provide the optimal neurotrophic effect to treat neurodegenerative disorders is of great clinical interest. Human UCB is an easily obtainable and rich source of relatively young SPCs. Therefore, we investigated the expression of the neurotrophic factors BDNF, NGF, NT-3, NT-4, GDNF, CDNF, MANF, and PEDF, as well as VEGF and the NT receptors p75NTR, TrkA, TrkB, TrkC, GFRα1, and GFRα2 in SPC-enriched populations isolated from human UCB under steady-state conditions and determined whether incubation in serum-free conditions affects their ability to express NTs in vitro. Additionally, we assessed whether SPC-conditioned medium could influence neural cell survival or proliferation. To investigate the ability of SPCs to synthesize, produce, and secrete proteins and determine their migratory capacities, proliferative potential, and differentiation capacity (features that may lead to a neuroprotective effect), we performed microarray gene expression profiling on NCs, lineage-negative, CD34^+^, and CD133^+^ cells following the isolation of these subpopulations. Our data reveal that these SPC populations are characterized by different expression patterns.

## Materials and Methods

### Sample collection

Human UCB samples were obtained from the Department of Pathological Obstetrics and Delivery of the Pomeranian Medical University in Szczecin, Poland. UCB samples were obtained from the placentas of healthy full-term neonates. This study adhered to the tenets of the Declaration of Helsinki, and approval was obtained from the Local Research Ethics Committee. Moreover, the women involved gave written informed consent prior to their involvement.

### Isolation of human cord blood lineage-negative, CD34^+^ and CD133^+^ cells

Umbilical cord blood samples were lysed in BD PharmLyse Lysing Solution (BD Biosciences, San Jose, CA, USA) for 15 min at room temperature in the dark and washed twice in phosphate–buffered saline (PBS). The obtained suspension of UCB nucleated cells (NCs) was subjected to immunomagnetic separation procedures. Each cell population, lineage-negative SPCs, CD34^+^ or CD133^+^ cells enriched in SPCs were isolated from non-separated NCs using immunomagnetic isolation and a Lineage Cell Depletion Kit, CD34 MicroBead Kit or CD133 MicroBead Kit (Miltenyi Biotec, Auburn, CA, USA). Isolation procedures were performed according to the manufacturer's instructions. Briefly, lineage-negative cells were isolated through negative selection using a MidiMACS separator (Miltenyi Biotec, Auburn, CA, USA). To isolate a lineage-negative cell population, a Lineage Cell Depletion Kit (Miltenyi Biotec, Auburn, CA, USA) was used. One hundred microliters of biotin-antibody cocktail recognizing the lineage-specific cell antigens were added per 10^8^ cells according to the manufacturer's recommendations. After washing in PBS, 100 µL of anti-biotin MicroBeads for magnetic cell labeling was added. Labeled cell suspension was loaded onto a MACS LS column (Miltenyi Biotec), and unlabeled cells passing through the column were collected (lineage-negative population).

CD34^+^ and CD133^+^ cells were isolated through positive selection using a MidiMACS separator (Miltenyi Biotec). To label CD34^+^ cells, a CD34 MicroBead Kit was used, and CD133^+^ cells were labeled using the CD133 MicroBead Kit (Miltenyi Biotec). One hundred microliters of FcR blocking reagent, which inhibits nonspecific or Fc-receptor-mediated binding, and 100 µL of CD34/CD133 microbeads for magnetic cell labeling were added per 10^8^ cells according to the manufacturer's recommendations. Labeled cell suspensions were subjected to immunomagnetic separation, in which magnetically labeled cells were retained in the LS MACS affinity column (Miltenyi Biotec) and unlabeled cells passed through the column. After several washes, the column was removed from the magnet, and the retained CD34^+^ or CD133^+^ cells were eluted with 1-5 mL of PBS supplemented with 0.5% bovine serum albumin and 2 mM EDTA.

From UCB units with relatively high volumes (at least 450×10^9^ NCs), we simultaneously isolated three SPC populations immunomagnetically, but from units with a lower UCB volume, we only isolated lineage-negative SPCs for a more detailed analysis. The isolation procedures were performed immediately after obtaining NCs from the UCB units. From the 56 UCB units processed, lineage-negative cells were isolated 56 times, and CD34^+^ and CD133^+^ cells were isolated 25 times. Total numbers of isolated cells were determined using a TC Automated Cell Counter (Bio-Rad Inc., Philadelphia, PA, USA).

### Flow cytometry

Human UCB was lysed in BD PharmLyse Lysing Solution (BD Biosciences, San Jose, CA, USA) for 15 min at room temperature and washed twice in PBS. The obtained suspension of UCB NCs was subjected to immunomagnetic separation procedures. Lineage-negative SPCs were enriched using a Lineage Cell Depletion Kit (Miltenyi Biotec, Auburn, CA, USA) according to the manufacturer's instructions. After isolation, lineage-negative cells were resuspended in 100 µL PBS and stained with mouse anti-human monoclonal antibodies for the following SPC markers: CD34 conjugated with allophycocyanin (APC) (BD Biosciences, San Jose, CA, USA), CD133 conjugated with phycoerythrin (PE) (Miltenyi Biotec, Auburn, CA, USA), and CD144 (FITC) (BD Biosciences, San Jose, CA, USA). Because the presence of the chemokine receptor CXCR4 is a major molecular signature of SPCs, lineage-negative cells were stained for the SPC marker CD34 conjugated with PE as well as CXCR4 conjugated with APC (both from BD Biosciences, San Jose, CA, USA). Additionally, we performed staining for mesenchymal stem cells (MSCs) within lineage-negative SPC population. Lineage-negative cells were stained using a Human MSC Analysis Kit (BD Biosciences, San Jose, CA, USA) according to the manufacturer's instructions. Cells were resuspended in 100 µL of PBS and stained with mouse anti-human monoclonal antibodies for the following markers: CD34, CD11b, CD19, CD45, PE-conjugated HLA-DR, FITC-conjugated CD90, PerCP-Cy5.5-CD105, and APC-conjugated CD73. After incubation for 20 min at 4°C, the cells were washed twice in PBS. Cell fluorescence was measured, and the data were analyzed using a fluorescence-activated cell analyzer (LSRII, BD Biosciences, San Jose, CA, USA) and BD FACSDiva software. Typically, 10,000 events were acquired to determine the percentage of a subpopulation within the lineage-negative SPCs. For better analysis of the MSCs markers, we acquired 100,000 events. Populations of hematopoietic SPCs based on CD34^+^ and CD133^+^ expression, early CD34^+^/CD133^+^/CD144^+^ endothelial progenitor cells (EPCs), endothelial CD144^+^ cells, and CD34^+^/CXCR4^+^ cells were analyzed.

After isolation, CD34^+^ and CD133^+^ cells were resuspended in 100 µL of PBS and stained with mouse anti-human monoclonal antibodies raised against the following SPC markers: PE-conjugated CD34 (BD Biosciences, San Jose, CA, USA), APC-conjugated CD133 (Miltenyi Biotec, Auburn, CA, USA), and FITC-conjugated CD144 (BD Biosciences, San Jose, CA, USA). Additionally, CD34^+^ and CD133^+^ cells were stained for the CXCR4 surface marker using an APC-conjugated antibody (both from BD Biosciences, San Jose, CA, USA). The cells were stained with DAPI and resuspended in 1% paraformaldehyde. After a 20-min incubation at 4°C, cells were washed twice with PBS. Fluorescence was measured, and the data were analyzed using a fluorescence-activated cell analyzer (LSRII, BD Biosciences, San Jose, CA, USA) and the BD FACSDiva software. The percent of cells expressing surface markers alone and co-expression was analyzed.

### Real time QRT-PCR

Total mRNA was isolated from NCs, lineage-negative cells, CD34^+^ cells, and CD133^+^ cells using the RNeasy Mini Kit (Qiagen, Valencia, CA, USA). Subsequently, mRNA was reverse-transcribed using the First Strand cDNA Synthesis Kit (Fermentas International Inc., Burlington, ON, Canada). Quantitative assessment of NTs and neurotrophin receptor (NTR) mRNA levels was performed using real time QRT-PCR carried out on a Bio-Rad CFX96 Real-Time PCR Detection System (Bio-Rad Inc., Philadelphia, PA, USA). The 25-µL reaction mixture contained 12.5 µL of SYBR Green PCR Master Mix, 10 ng of cDNA template, and one pair of forward and reverse primers. The primers used for these reactions are listed in Table 1. The real-time cycling conditions were as follows: 1 cycle at 95°C for 10 min, followed by 40 cycles at 95°C for 15 sec, 60°C for 1 min, and 72°C for 15 sec. Relative target gene mRNA expression was quantified using the comparative Ct method. The relative quantification value of the target was normalized to the endogenous control beta-2 macroglobulin (BMG) gene and expressed as 2ΔCt, where ΔCt  =  [Ct of target genes] - [Ct of endogenous control gene (BMG)].

**Table 1:** The primers used for real time QRT-PCR reactions.

BDNF	f. 5′ GATGCTCAGTAGTCAAGTGCC 3′
	r. 5′ GCCGTTACCCACTCACTAATAC 3′
NGF	f. 5′ GCAAGCGGTCATCATCCCAT 3′
	r. 5′ TGTTGTTAATGTTCACCTCTCCC 3′
NT-3	f. 5′ GGTACGCGGAGCATAAGAGTC 3′
	r. 5′ GAGAGTTGCCCGTTTTGATCT 3′
NT-4	f. 5′ GCGAGGTGGAGGTGTTGG 3′
	r. 5′ CCTTCCTCAGCGTTATCAGC 3′
GDNF	f. 5′ TGGCAGTGCTTCCTAGAAGAG 3′
	r. 5′ AAGACACAACCCCGGTTTTTG 3′
VEGF	f. 5′ GAGCCGCGAGAAGTGCTA 3′
	r. 5′ GCCTCACCCGTCCATGAG 3′
CDNF	f. 5′ CAGTTGGCCGTCAGTTTTG 3′
	r. 5′ ACCACTGCATCCTCCAAGAA 3′
MANF	f. 5′ CTGGGACGATTTTACCAGGA 3′
	r. 5′ CCGATTCTCTTTGCCTCTTG 3′
PEDF	f. 5′ ACCTGATCACCAACCCTGAC 3′
	f. 5′ GCCTGCACCCAGTTGTTAAT 3′
p75NTR	f. 5′ CCTACGGCTACTACCAGGATG 3′
	r. 5′ CACACGGTGTTCTGCTTGTC 3′
TrkA	f. 5′ GTCAGCCACGGTGATGAAATC 3′
	r. 5′ CAGCACGTCACGTTCTTCCT 3′
TrkB	f. 5′ CTGGTGCATTCCATTCACTG 3′
	r. 5′ CGTGGTACTCCGTGTGATTG 3′
TrkC	f. 5′ TGGCTGGACTATGTGGGCT 3′
	r. 5′ CCCATTGCTGTTCCCTGAATC 3′
GFRα1	f. 5′ GTACAGGTCGGCGTACATCAC 3′
	r. 5′ AGCAGAAGAGCATTCCGTAGC 3′
GFRα2	f. 5′ AGCGCCAAGAGCAACCATT 3′
	r. 5′ CATGCGGTAGGTGTACTCGA 3′
Bax	f. 5′ GTT GCG GTC AGA AAA CAT GTC 3′
	r. 5′ GCC GCC GTG GAC ACA 3′
Bcl2	f. 5′ GCC GGT TCA GGT ACT CAG TCA T 3′
	r. 5′ CAT GTG TGT GGA GAG CGT CAA 3′
Bcl-xL	f. 5′ TCC CTC AGC GCT TGC TTT AC 3′
	r. 5′ GCC ACA GCA GCA GTT TGG 3′
BMG	f. 5′ AATGCGGCATCTTCAAACCT 3′
	r. 5′ TGACTTTGTCACAGCCCAAGATA 3′

### Western blot analysis

Western Blot analysis was performed on extracts prepared from UCB cell populations (NCs, CD34^+^, CD133^+^, lineage-negative) (3×10^6^) and then the cell lysates were collected and analyzed in order to evaluate the NTs and NT receptor expression. The cell pellets were lysed for 10 min on ice in M-Per lysing buffer (Pierce, Rockford, IL) containing protease and phosphatase inhibitors (Sigma-Aldrich, St. Louis, MO, USA): 10 µg/ml leupeptin, 10 µg/ml aprotinin, 1 µg/ml pepstatin A, 1 mM sodium fluoride, and 2 mM Na3VO4). Cell lysates were clarified by centrifugation at 14,000 rpm for 10 min at 4°C, and the protein concentrations were determined using the Bradford protein assay (Sigma-Aldrich, St. Louis, MO, USA). Equal amounts of protein (20 µg/well) were loaded and separated on 4–20% sodium dodecyl sulfatepolyacrylamide gel electrophoresis (SDS-PAGE, mini-PROTEAN II electrophoresis system, Bio-Rad Inc., Philadelphia, PA, USA) and then transferred to a 0.2 µm polyvinylidene fluoride (PVDF) membrane (Bio-Rad Inc., Philadelphia, PA, USA). Kaleidoscope polypeptide standard wide range (10 to 250 kD) protein markers (Bio-Rad Inc., Philadelphia, PA, USA) were used to determine the molecular weights of the NTs and NT receptors. Following blockage 2 h at room temperature with 3% BSA, TRIS-HCL and NaCl solution with 0.05% Tween 20, the membrane was probed with a specific monoclonal/polyoclonal IgG antibody directed against amino acids sequence of selected proteins (BDNF, GDNF, NGF, NT-3, NT-4, TrkA, TrkB, TrkC, p75NTR, BMG): rabbit anti-BDNF polyclonal antibody (at 1∶600 dilution), goat anti-GDNF polyclonal antibody (at 1∶600 dilution), rabbit anti-NGF polyclonal antibody (at 1∶600 dilution), goat anti-NT3 polyclonal antibody (at 1∶600 dilution), mouse anti-NT4 monoclonal antibody (at 1∶500 dilution), rabbit anti-TrkA polyclonal antibody (at 1∶1000 dilution), goat anti-TrkB polyclonal antibody (at 1∶500 dilution), rabbit anti-TrkC polyclonal antibody (at 1∶500 dilution), mouse anti-p75NGFR monoclonal antibody (at 1∶1000 dilution), rabbit polyclonal antibody anti-GFRA1 (at 1∶750 dilution), and mouse anti-BMG monoclonal antibody (at 1∶2000 dilution) (Santa Cruz Biotechnology, Santa Cruz, CA, USA, except anti-TrkA from Cell Signaling Technology, Inc. Danvers, MA, USA) and incubated overnight at 4°C. Immunoreactive bands were detected using horseradish peroxidase-conjugated secondary Ab (Santa Cruz Biotechnology, Santa Cruz, CA, USA) specific for primary antibody which were used in the previous step of analysis. Chemiluminescence detection was performed using the ECL Advance Detection Kit (Amersham Life Sciences, Buckinghamshire, UK) and bands were subsequently visualized in UVP camera (Gel DOC-It Imaging system; Bio-Rad Inc., Hercules, CA, USA). Equal loading in the lanes was evaluated by stripping the blots for 2 h at 37°C and then overnight at room temperature (IgG Elution Buffer; Thermo Scientific, Rockford, IL, USA) and reprobing them in analogous way with BMG monoclonal IgG Ab and then secondary HRP-conjugated antibody.

### Immunofluorescent analysis

SPC populations from UCB were isolated using the Lineage Cell Depletion Kit or the CD34 or CD133 MicroBead Kits (all from Miltenyi Biotec, Auburn, CA, USA) according to the manufacturer's instructions. Isolated SPCs were subjected to immunofluorescence (IF) staining of neurotrophic factors. Briefly, lineage-negative, CD34^+^, or CD133^+^ SPCs were fixed in 3.5% paraformaldehyde for 20 min, permeabilized using 0.1% Triton X-100 for 10 min, washed in PBS, and subsequently stained with primary antibody (ab). To measure the expression of BDNF or NGF, cells were incubated with rabbit anti-BDNF (Santa Cruz Biotechnology, Santa Cruz, CA, USA) or rabbit anti-NGF (Santa Cruz Biotechnology, Santa Cruz, CA, USA) for 1 h at room temperature. Next, the cells were incubated with the secondary ab goat anti-rabbit Texas Red (Vector Laboratories, Inc., Burlingame, CA, USA) for 1 h at room temperature in the dark. To measure the expression of NT-3 or GDNF, cells were incubated with goat anti-NT-3 (Santa Cruz Biotechnology, Santa Cruz, CA, USA) or goat anti-GDNF for 1 h at room temperature and subsequently incubated with the secondary ab donkey anti-goat AlexaFluor 647 (Life Technologies, Paisley, UK) for 1 h at room temperature in the dark. For NT-4 expression, cells were incubated with mouse anti-NT-4 (Santa Cruz Biotechnology, Santa Cruz, CA, USA) for 1 h at room temperature. The cells were then incubated with the secondary ab goat anti-mouse FITC (BD Pharmingen, San Diego, CA, USA) for 1 h at room temperature in the dark. Next, the presence of neurotrophin receptors in lineage-negative, CD34^+^ or CD133^+^ SPCs was also confirmed using IF staining. To examine the coexpression of p75NTR and Trka receptors, cells were incubated with rabbit anti-p75NTR (Bioss, Inc., Woburn, MA, USA) for 1 h at room temperature and then incubated for 1 h at room temperature in the dark with the secondary abs goat anti-rabbit Texas Red (Vector Laboratories, Inc., Burlingame, CA, USA) and mouse anti-TrkA conjugated with APC (R&D Systems, Minneapolis, MN, USA). To study the coexpression of p75NTR and TrkB, cells were incubated with rabbit anti-p75NTR (Bioss, Inc., Woburn, MA, USA) and mouse anti-TrkB (R&D Systems, Minneapolis, MN, USA) for 1 h at room temperature and subsequently incubated with the secondary abs goat anti-rabbit Texas Red (Vector Laboratories, Inc., CA, USA) and anti-mouse FITC (BD Pharmingen, San Diego, CA, USA) respectively for 1 h at room temperature in the dark. To study the coexpression of the p75NTR and TrkC receptors, cells were incubated with rabbit anti-p75NTR (Bioss, Inc., Woburn, MA, USA) and mouse anti-TrkC (R&D Systems, Minneapolis, MN, USA) for 1 h at room temperature and subsequently incubated for 1 h at room temperature in the dark with the secondary abs goat anti-rabbit Texas Red (Vector Laboratories, Inc., Burlingame, CA, USA) and anti-mouse FITC (BD Pharmingen, San Diego, CA, USA). Nuclei were stained with DAPI (BD Pharmingen, Franklin Lakes, NJ, USA) for 10 min in the dark. Fluorescent images were captured using the Pathway Bioimager System (BD Bioscences, Rockville, MD, USA).

### Incubation in serum-free conditions

NCs or lineage-negative SPCs were incubated at a density of 1.5 to 2.5×10^6^ cells/well in a 24-well culture plate on non-treated plastic in serum-free Iscove's Modified Dulbecco's Medium (IMDM) (Sigma-Aldrich, St. Louis, MO, USA) containing albumin (5%), penicillin (100 U/mL), streptomycin (100 ug/mL), and L-glutamine (2 mM) at 37°C under a humidified atmosphere containing 5% CO_2_. Cells were collected after 24, 48, and 72 h incubations. Incubated cells were subjected to total number analysis, total mRNA isolation, and protein isolation at both 24, 48, and 72 h. Total cell numbers were analyzed using a TC Automated Cell Counter (Bio-Rad Inc., Philadelphia, PA, USA). RT-PCR and western blots of cells obtained under steady-state conditions and after serum-free incubation were compared. Similarly, populations of NCs or lineage-negative SPCs were incubated in Iscove's Modified Dulbecco's Medium (IMDM) (Sigma-Aldrich, St. Louis, MO, USA) containing bovine calf serum (5%), penicillin (100 U/mL), streptomycin (100 ug/mL), and L-glutamine (2 mM) at 37°C under a humidified atmosphere containing 5% CO_2_. After incubation, the cells were subjected to the procedures previously described above.

### RNA Isolation and Affymetrix GeneChip Microarray and data analysis

Total RNA was isolated from human UCB-derived cell populations using an RNeasy Mini Kit (Qiagen, Valencia, CA, USA). RNA isolates from three separate cell isolations per SPC population (NCs, lineage-negative, CD34^+^, and CD133^+^) were pooled to generate four samples for subsequent experimental procedures. Sense-strand cDNA generated from total RNA using an Ambion WT Expression Kit (Life Technologies, Paisley, UK) was fragmented and labeled using the GeneChip® WT Terminal Labeling Kit (Affymetrix, Santa Clara, CA, USA) and hybridized onto an Affymetrix WT Array Strip. Hybridization as well as subsequent fluidics and scanning steps were performed using an Affymetrix GeneAtlas™ system (Affymetrix, Santa Clara, CA, USA). Microarray data are available in the ArrayExpress database (www.ebi.ac.uk/arrayexpress) under accession number E-MTAB-2053. The differences in expression of the chosen genes and Gene Ontology terms were analyzed in the R programming environment using Bioconductor packages.

### Lineage-negative conditioned medium (CM) preparation

Lineage-negative cells were obtained from UCB as described above. Cells were subsequently seeded at a density of 1.5 to 2.5×10^6^ cells/well in a non-treated 24-well plastic culture plate in serum-free IMDM with 5% albumin, penicillin (100 U/mL), streptomycin (100 µg/mL), and L-glutamine (2 mM) and incubated for 24 h at 37°C in a humidified atmosphere containing 5% CO_2_. Then, conditioned medium (CM) was harvested. The CM aliquots were pooled and concentrated 10 times by centrifugation at 4000×*g* for 20 min at room temperature using 10-kDa MW cut-off filter units (Merck Millipore, Billerica, MA, USA). Filtrate volumes of approximately 1000 µL were consistently obtained from 10 mL starting volumes. Concentrated CM were stored at -80°C until use.

### Cell culture

SH-SY5Y neuroblastoma cells (human, ECACC; Sigma Aldrich, St. Louis, MO, USA) were maintained in 1∶1 Dulbecco's modified Eagle's medium/Ham's F-12 medium supplemented with 1% non-essential amino acids, 2 mM L-glutamine, antibiotics (100 IU/mL penicillin and 100 µg/mL streptomycin), and 10% fetal bovine serum (FBS). All cultures were maintained at 37°C with 5% CO_2_ and 100% humidity. The medium was replaced every three days, and cells were divided into separate flasks before reaching confluence.

### Lineage-negative conditioned medium-treated SH-SY5Y human neuroblastoma cells

Prior to incubation with lineage-negative conditioned medium, SH-SY5Y cells were seeded at a density of 3×10^5^ cells/well in a 6-well culture plate or at a density of 5×10^3^ cells/well on poly-D-lysine-coated 8-well culture slides in culture medium (1∶1 Dulbecco's modified Eagle's medium/Ham's F-12 medium supplemented with 1% non-essential amino acids, 2 mM L-glutamine, antibiotics [100 IU/mL penicillin and 100 µg/mL streptomycin], and 10% FBS). Twenty-four hours after seeding, the culture medium was replaced, and SH-SY5Y cells were incubated for 24 h in serum-free culture medium supplemented with 25% lineage-negative CM. As a control, we used serum-free culture medium supplemented with 25% Iscove's Modified Dulbecco's Medium with 5% albumin. All cultures were maintained at 37°C with 5% CO_2_ and 100% humidity.

### Human neuroblastoma cell proliferation

#### BrdU incorporation

Cell proliferation was assessed by measuring BrdU incorporation into newly synthesized DNA strands of actively proliferating cells using an Apoptosis, DNA damage, and Cell Proliferation Kit according to the manufacturer's protocol (BD Biosciences, San Jose, CA, USA). Specifically, SH-SY5Y cells were seeded at a density of 3×10^5^ cells/well in a 6-well culture plate. Twenty-four hours after seeding, SH-SY5Y cells were treated for 24 h with one of the following serum free media conditions to assess proliferation: (1) culture medium + 25% lineage-negative CM or (2) culture medium + 25% IMDM medium with 5% albumin. All cultures were maintained at 37°C with 5% CO_2_ and 100% humidity. Cells were incubated with a 10-µM BrdU labeling solution at 37°C for 2 h and then trypsinized. BrdU incorporation was measured by flow cytometry using a FITC-conjugated mouse monoclonal anti-BrdU antibody. The experiment was repeated three times. The results are expressed as the mean percentage of cells in S phase.

### Ki-67 expression

Cell proliferation was analyzed by immunofluorescent staining with a monoclonal antibody raised against Ki-67. Ki-67 is a human nuclear protein whose expression is strictly associated with cell proliferation. SH-SY5Y cells were seeded at a density of 5×10^3^ cells/well on poly-D-lysine-coated 8-well culture slides. Twenty-four hours after seeding, SH-SY5Y cells were treated for 24 h with one of the following serum free media conditions to assess proliferation: (1) culture medium + 25% lineage-negative CM or (2) culture medium + 25% IMDM medium with 5% albumin. Cultures were maintained at 37°C with 5% CO_2_ and 100% humidity. After 24 h, cells were fixed with 4% paraformaldehyde (PFA) for 1 h at room temperature. To visualize the cell membrane, slides were incubated with wheat germ agglutinin (WGA) conjugated to Alexa Fluor 647 (Life Technologies, Paisley, UK). After permeabilization with 0.25% Triton X-100 (Sigma Aldrich, MO, USA) and blocking in 10% normal goat serum, sections were incubated with a primary rabbit anti-Ki67 antibody (1∶50) (Abcam, Cambridge, UK) in PBS supplemented with 1% BSA at 4°C overnight. Next, the sections were incubated with a goat anti-rabbit Texas Red secondary antibody (1∶100) (Vector Laboratories, Burlingame, CA, USA) for 1 h at room temperature protected from light. Then, the cells were counterstained with a DAPI solution (Thermo Scientific, Pittsburgh, PA, USA). After staining, slides were detached from their chambers, mounted, and subjected to microscopy analysis using a LSM700 confocal system (Carl Zeiss, Jena, Germany).

### Assessment of human neuroblastoma cell apoptosis

Detection and quantification of apoptosis were performed by analyzing the apoptotic gene expression of Bax, Bcl-2, and Bcl-xL. SH-SY5Y cells were seeded at a density of 3×10^5^ cells/well in 6-well culture plates. Twenty-four hours after seeding, SH-SY5Y cells were treated for 24 h with one of the following serum-free media: (1) culture medium + 25% lineage-negative CM or (2) culture medium + 25% Iscove medium with 5% albumin. The cells were trypsinized, and total RNA was isolated as described above. Apoptotic gene expression was evaluated by qRT-PCR. Primers used in PCR reactions are listed in Table 1. The results are expressed as the mRNA expression of apoptotic genes.

### Statistics

Because the distribution of most variables significantly deviated from normal distribution, non-parametric tests were used. The significance of differences between the SPC-enriched populations and between parameters that were measured in the steady-state and after 24, 48 or 72 h incubations of SPCs was assessed using the Kruskal-Wallis test followed by the Mann-Whitney test. P values<0.05 were considered statistically significant.

## Results

### Human UCB-derived SPCs

Characterizing human SPCs is crucial for advancing cell biology and cell-based therapies. Clinical and basic science studies have suggested that neurotrophic factor secretion plays several pathophysiological roles in neural tissue regeneration, and it has been found that human UCB-derived cells produce NTs [13]. Because UCB had been described as a potential source for SPCs with relatively immature characteristics [22], we were interested in whether selected UCB-derived SPC populations could be a source of neurotrophic factors for use in neuroregeneration. Thus, we employed a strategy analogical to hematopoietic stem-cell transplantation to isolate SPC populations from UCB and subsequently used these cells in our research to specifically investigate human UCB-derived lineage-negative, CD34^+^, and CD133^+^ cells. We selected lineage-negative SPCs, CD34^+^ cells, and CD133^+^ cells because these cells have been widely used in clinical applications, particularly in hematology, as well as for experimental studies concerning neuroregeneration. Non-separated UCB-derived nucleated cells (NCs) were used as the control.

The mean volume of collected UCB units was 61.62±23.3 mL and ranged from 32 to 137 mL. The total average number of NCs recovered from a single unit was 473.7±287.54×10^6^ cells. We successfully isolated the three SPC-enriched populations from human UCB using immunomagnetic separation. The number of cells recovered from 100×10^6^ NCs for each SPC population was 1.68±1.35×10^6^, 1.25±0.78×10^6^, and 1.04±0.81×10^6^ for lineage-negative, CD34^+^, and CD133^+^ cells, respectively. Accordingly, in our studies, the UCB-derived NC fraction contained 1.68±1.35% lineage-negative SPCs, 1.25±0.78% CD34^+^cells, and 1.04±0.81% CD133^+^ cells.

### Lineage-negative, CD34+, and CD133+ cells are heterogeneous cell populations expressing SPC markers

There is considerable interest in utilizing UCB-derived lineage-negative cells that are highly enriched for immature SPCs in cell therapy for neurologic disorders. However, lineage-negative cells are a very heterogeneous population and are therefore not well characterized. By employing flow cytometry, we assessed the percentage of cells expressing stem/progenitor -specific surface markers among the lineage-negative cell population that was isolated using an immunomagnetic separation procedure. We found that the proportion of expressed markers in lineage-negative cells reflects their immaturity, such that CD34^+^ was expressed in 12.1±7.2% of cells and that CD133^+^ was expressed in 12.3±8.2% of cells. The lineage-negative SPCs also expressed a marker known to be involved in SPC migration, adhesion, and homing to the bone marrow and sites of tissue injury, i.e., the SDF-1 cognate receptor CXCR4 (89.9±3.3%). In contrast, lineage-negative cells exhibited a low percentage of early CD34^+^/CD133^+^/CD144^+^ EPCs (1.7%). Because mesenchymal stem cells have been shown to exist in umbilical cord blood [23], lineage-negative cells were analyzed to evaluate the percentage of cells possessing characteristic mesenchymal stem cell markers. As shown in Table 2, cells with a MSC phenotype of CD105^+^/CD73^+^/CD90^+^/CD45^−^/CD34^−^/CD11b^−^/CD19^−^/HLA-DR^−^ are extremely rare within the lineage-negative population (0.0084%±0.0108%). The percentage data are shown in Table 2. Together, these results demonstrate that lineage-negative cell populations constitute primitive hematopoietic and endothelial stem cells while simultaneously containing other immature SPCs.

**Table 2:** Phenotypic characterization of immunomagnetically isolated stem/progenitor cell populations of UCB.

Antigen	Cells expressed stem/progenitor surface markers among human UCB-derived		
	lineage-negative cells	CD34^+^ cells	CD133^+^ cells
	(mean ± SD)	(mean ± SD)	(mean ± SD)
CD34^+^	12.1%±7.2%	91.2%±4.8%	60.3%±9.2%
CD133^+^	12.3%±8.2%	72.1%±12.6%	94.2%±5.1%
CD34^+^/CD133^+^	8.5%±5.0%	69.5%±11.3%	55.43%±13.4%
CXCR4^+^	89.9%±3.3%	74.9%±13.1%	50.4%±11.0%
CD144^+^	3.1%±1.6%	5.5%±1.8%	4.9%±2.1%
Endotelial progenitor cells (CD34^+^/CD133^+^/CD144^+^)	1.7%±1.1%	3.8%±2.1%	3.3%±1.4%
Mesenchymal stem cells (CD105^+^/CD73^+^/CD90^+^/CD45^−^/CD34^−^/CD11b^−^/CD19^−^/HLA-DR^−^)	0.0084%±0.0108%	—	—

Similarly, isolated CD34^+^ cells were analyzed by flow cytometry to evaluate the expression of hematopoietic- and endothelial-associated surface antigens. As presented in Table 2, CD34 was expressed in 91.2±4.8% of immunomagnetically isolated UCB CD34^+^ cells, and the other analyzed antigens were expressed as follows: CD133, 72.1±12.6%; CXCR4, 74.9±13.1%; and CD144, 5.5±1.8%. Furthermore, the co-expression of CD34, CD133, and CD144 surface antigens on UCB CD34^+^ cells was evaluated. Nearly 70% of the UCB CD34^+^ cells were CD34^+^/CD133^+^ (69.5±11.3%), and 3.8±2.1% of the cells were CD34^+^/CD133^+^/CD144^+^. Next, to determine the expression of hematopoietic- and endothelial-associated surface antigens on immunomagnetically isolated CD133^+^ cell population, a similar evaluation was performed. Accordingly, the CD133 antigen was expressed in 94.2±5.1% of isolated UCB CD133^+^ cells, and other antigens were expressed as follows: CD34, 60.3±9.2%; CXCR4, 50.4±11.0%; and CD144, 4.9±2.1% (Table 2). Co-expression analysis of the CD34, CD133, and CD144 antigens on UCB CD133^+^ cells revealed that the majority of UCB CD133^+^ cells were CD34^+^/CD133^+^ (55.43±13.4%), but 3.3±1.4% cells were CD34^+^/CD133^+^/CD144^+^. These findings clearly indicate that both CD34^+^ and CD133^+^ are heterogeneous cell populations containing cells that express different markers characteristic of hematopoietic and endothelial SPCs.

### UCB SPC populations express NTs under steady-state conditions

**Figure 1:**
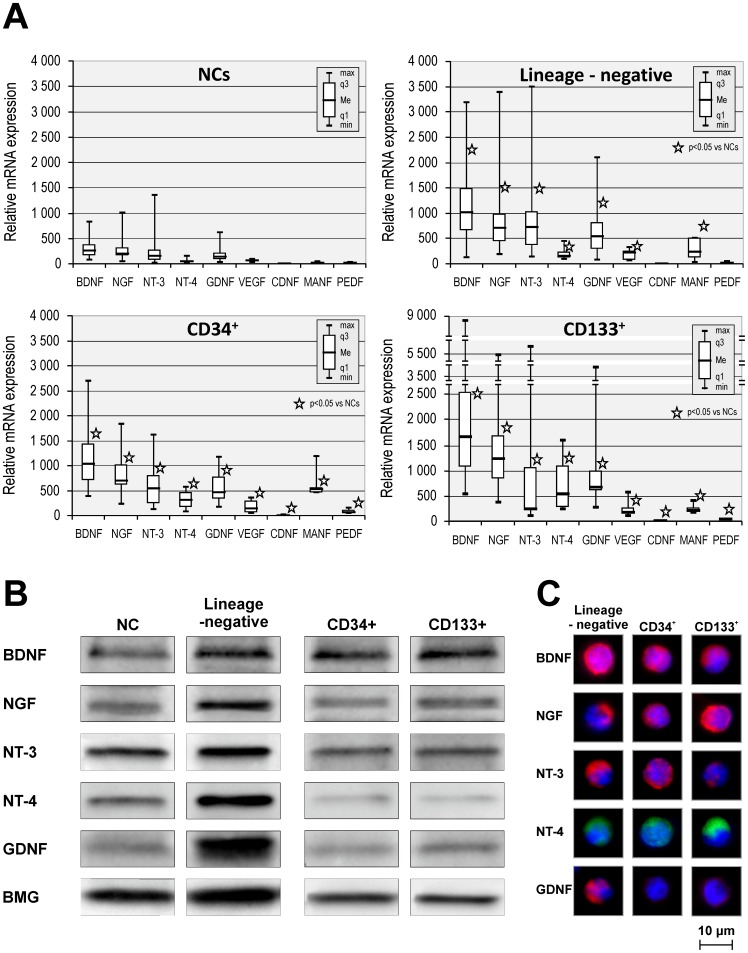
UCB SPC populations continuously express neurotrophins (NTs). (A) NT mRNA expression levels in UCB-derived nucleated cells, lineage-negative, CD34^+^, and CD133^+^ cells. Relative of mRNA expression values are normalized against BMG expression values. Data are presented as median, quartiles, interquartile range, minimum, and maximum for six independent experiments. *P<0.05 versus NCs (the exact statistical difference is presented in the Results). (B) Representative western blot analysis of NT protein levels in SPC lysates. (C) Immunofluorescent analysis of intracellular NT expression in SPCs. Nuclei were visualized by DAPI staining. Pseudo-coloring was assigned to each stain as follows: anti-BDNF, anti-NGF, anti-NT-3, and anti-GDNF are red; anti-NT-4 is green, and nuclei are blue. All images were captured using the Pathway Bioimager System (BD Biosciences, Rockville, MD, USA). Representative data are shown from three independent experiments.

Abundant evidence indicates that neuroprotective factors contribute neurogenic and angiogenic effects that are observed after UCB-derived cells are transplanted into the site of a CNS injury [10]. Accordingly, we evaluated the expression of various NTs and VEGF at the mRNA level in lineage-negative SPCs, CD34^+^, and CD133^+^ cells using QRT-PCR and compared these expression levels to those observed in unsorted NCs. We found that NTs and VEGF are robustly expressed at the mRNA level in SPCs. The expression data are shown in Fig. 1A. Univariate statistical analysis revealed that all assessed populations (lineage-negative SPCs, CD34^+^ cells, and CD133^+^ cells) expressed significantly higher levels of each neurotrophic factor (i.e., BDNF (p<0.0001), NGF (p<0.0001), NT-3 (p<0.05), NT-4 (p<0.0001), GDNF (p<0.0001), CDNF (p<0.05), MANF (p<0.05), PEDF (p<0.05), and VEGF (p<0.05)) than unsorted NCs. Of note, lineage-negative SPCs expressed higher levels of NT-3 than the CD133^+^ population (p<0.05). However, the CD133^+^ population expressed significantly higher levels of NGF (p<0.05), NT-4 (p<0.05), and CDNF (p<0.05) than lineage-negative SPCs. Quantitatively, the lineage-negative SPC population and the CD34^+^ population expressed NTs at a similar level but in a slightly different pattern.

Furthermore, we observed tendency toward increased expression of NTs in UCB-derived NCs, lineage-negative SPCs, CD34^+^, and CD133^+^ populations at the protein level as determined by Western blot analysis (Fig. 1B). Finally, we confirmed the expression of NTs by visualizing their content in isolated SPCs by performing immunofluorescent analyses. We determined that isolated SPC-enriched (lineage-negative, CD34^+^, and CD133^+^) populations expressed the selected NTs (i.e., BDNF, NGF, NT-3, NT-4, and GDNF) as shown by immunofluorescent staining (Fig. 1C). Collectively, we found that UCB-derived SPCs expressed all of the assessed NTs and VEGF at higher levels than unsorted NCs. This result may suggest that these cells contribute to the neurogenic and angiogenic effects seen in UCB-derived cells that are transplanted in the site of neural injury during experimental studies.

### UCB SPC populations consistently express neurotrophin receptors

In recent years, numerous studies have shown that UCB cell-induced neuroprotection involves neurotrophic factors acting by paracrine and/or autocrine interactions between the neural microenvironment and transplanted cells [13], [15], [24]. Studying receptor expression based on the unique SPC-enriched populations is critical to understanding the effects of trophic factors performing within the neurogenic niche where they are recruited or transplanted. Using QRT-PCR, we evaluated the expression levels of the neurotrophin receptors p75NTR, TrkA, TrkB, TrkC, GFRα1, and GFRα2 in SPC-enriched UCB-derived populations and compared them with the expression levels of these receptors in unsorted NCs.

**Figure 2:**
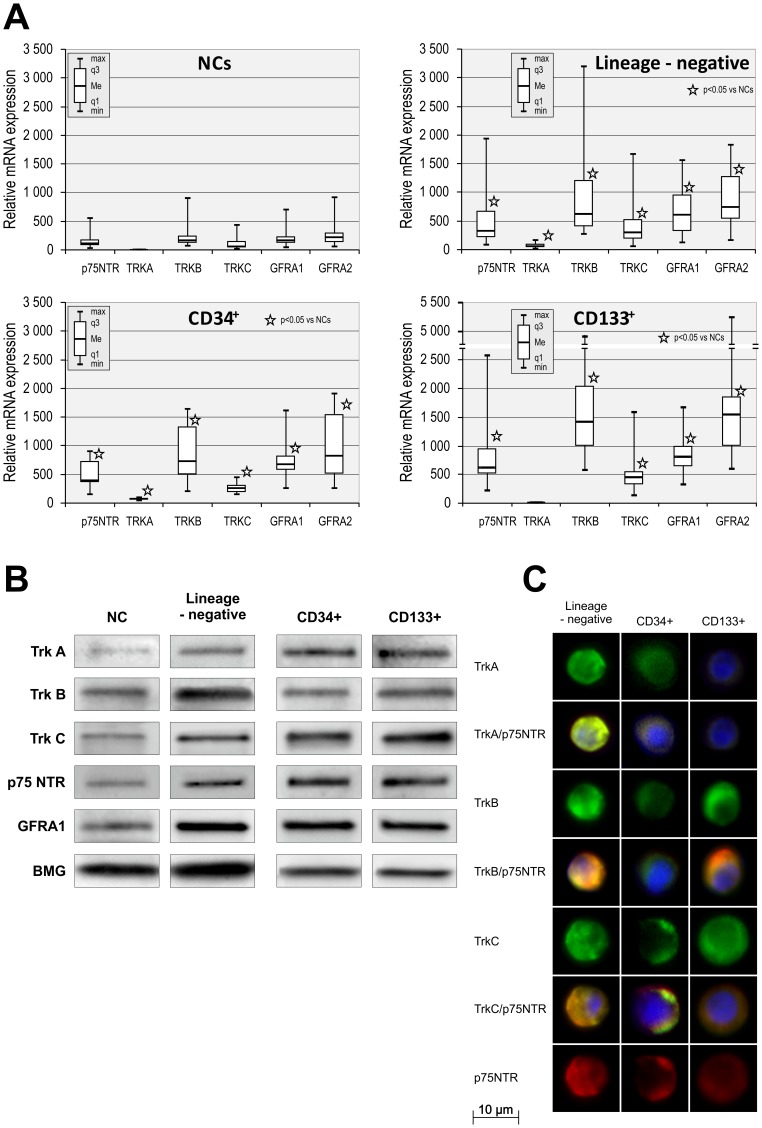
UCB SPC populations continuously express neurotrophin receptors. (A) NT receptor mRNA expression levels in UCB-derived nucleated cells, lineage-negative, CD34^+^, and CD133^+^ cell populations. Relative mRNA expression values are normalized against BMG levels. Data are shown as median, quartiles, interquartile range, minimum, and maximum for six independent experiments. *P<0.05 versus NCs (the exact statistical difference is presented in the Results). (B) Representative western blot analysis of NT receptor protein levels in SPC lysates. (C) Immunofluorescent analysis of NT receptor expression on the surface of SPCs. Nuclei were visualized by DAPI staining. Pseudo-coloring was assigned to each stain as follows: anti-TrkA, anti-TrkB, and anti-TrkC – green, anti-p75NTR – red, and nuclei – blue. All images were captured using the Pathway Bioimager System (BD Biosciences, Rockville, MD, USA). Representative data are shown from three independent experiments.

All neurotrophin receptors were expressed at detectable levels by each cell type as demonstrated in Fig. 2A. Univariate statistical analysis revealed that lineage-negative SPCs, CD34^+^ cells, and CD133^+^ cells expressed significantly higher levels of each neurotrophin receptor (p75NTR (p<0.0001), TrkA (p<0.05), TrkB (p<0.05), TrkC (p<0.0001), GFRα1 (p<0.0001), and GFRα2 (p<0.0001)) compared to unsorted NCs. In a statistical analysis comparing neurotrophin receptor expression among the SPC populations, lineage-negative SPCs expressed significantly lower levels of p75NTR (p<0.05), TrkA (p<0.0001), TrkC (p<0.05), and GFRα2 (p<0.05) receptors than CD133^+^ cells. Similarly, CD34^+^ cells expressed significantly lower levels of TrkA (p<0.0001) and TrkC (p<0.05) receptors than CD133^+^ cells. There were no statistically significant differences between the expression levels of NT receptors in lineage-negative SPCs and the CD34^+^ cell population.

Furthermore, we observed tendency toward the increased expression of almost all of the NT receptors in UCB-derived lineage-negative SPCs, CD34^+^, and CD133^+^ populations isolated from human UCB at the protein level, as determined using western blots (Fig. 2B). Finally, to visualize SPCs based on NT receptor expression, we isolated SPC-enriched populations from UCB and performed immunofluorescent analysis. We found that immunomagnetically isolated SPC-enriched populations (lineage-negative, CD34^+^, and CD133^+^) expressed the selected NT receptors p75NTR, TrkA, TrkB, and TrkC based on immunofluorescence staining (Fig. 2C). Furthermore, to confirm the coexpression of specific receptors with nonspecific p75NTR, we performed double-staining using antibodies against TrkA, TrkB or TrkC and p75NTR. Coexpression of these receptors was confirmed (Fig. 2C). Taken together, these results demonstrate the constitutive expression of specific NT receptors in SPC populations and suggest that these cells might respond to neurogenic stimulation in the CNS upon transplantation.

### Whole genome microarray analysis reveals large-scale alterations in gene expression between lineage-negative SPCs, CD34^+^ cells, and CD133^+^ cells

To further characterize the human UCB-derived SPCs, we analyzed the global gene expression pattern in the lineage-negative, CD34^+^, CD133^+^ cell populations. Microarray analysis revealed that 146 genes were at least 4-fold upregulated in lineage-negative cells compared to CD34^+^ cells, and 121 genes were upregulated by 4-fold or greater in lineage-negative cells when compared to CD133^+^ cells. The ten upregulated genes with the largest change in expression are presented in Table S1 for the lineage-negative SPCs compared to the CD34^+^ population, and in Table S2 for the lineage-negative SPCs compared to the CD133^+^ population. Similarly, our array analysis identified differences in gene expression between each of the analyzed SPC populations and NCs (data not shown).

**Figure 3:**
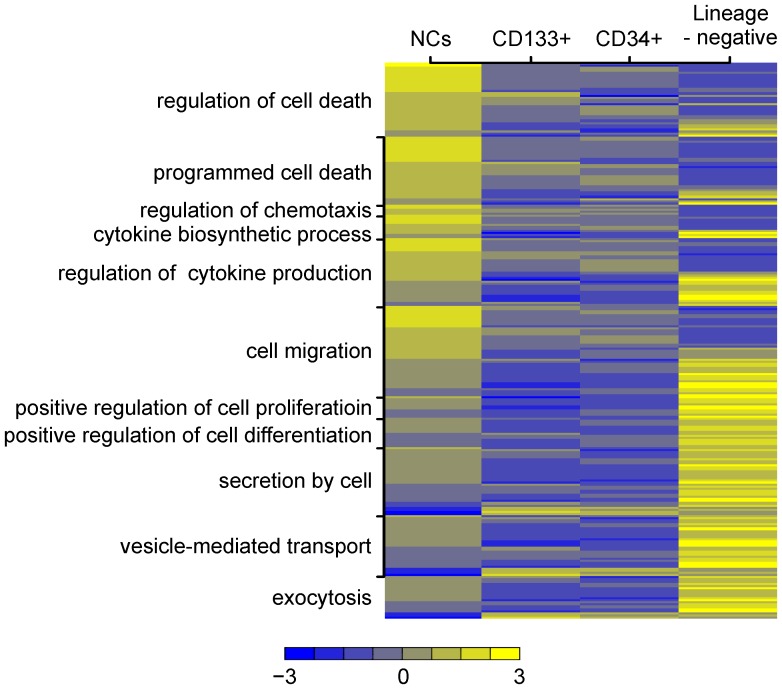
Global gene expression changes in human UCB SPCs (NCs, lineage-negative, CD34^+^, and CD133^+^ cells). The heatmap represents the expression levels of highly overexpressed genes (Fold Change >4). Individual genes are assigned according to the Gene Ontology classification of specific biological processes listed on the left side of the graph. Each column comprises a set of horizontal lines, each representing a single gene. Levels of gene expression are indicated on a color scale, with yellow corresponding to the highest level of expression and blue corresponding to the lowest level. The range of expression rate of the analyzed genes is shown below the graph. The diagram shows large-scale alterations in gene expression between NCs, lineage-negative SPCs, CD34^+^ cells, and CD133^+^ cells.

Next, all of the differentially expressed genes were classified according to the Gene Ontology (GO) Classification of Biological Processes. Functional analysis using GO revealed that a number of pathways were specifically and diversely represented in the analyzed UCB-derived SPC populations. Given the ability of SPCs to exert a neuroprotective effect via trophic action, we were interested in biological processes concerning biosynthetic processes, cytokine production, secretion by cells, chemotaxis, migration, and proliferative capacities. Comparing the bioinformatic analysis of the complex gene dataset in lineage-negative SPCs to that of CD34^+^ cells identified that genes involved in the regulation of cytokine production, secretion by cells, exocytosis, production of the tumor necrosis factor superfamily of cytokines, chemotaxis, cell migration, cell motility, regulation of chemotaxis, and the positive regulation of cellular differentiation were among the most upregulated (Table S3); these genes included the specific genes ITGB3, AZU1, PDGFA, CCL5, THBS1, GATA1, NPM1, and PDGFA. In lineage-negative SPCs compared to CD133^+^ cells, genes such as PDGFA, GATA1, CCR3, CSF1, RGCC, BAMBI, SEMA7A, BMP6, CLEC5A and CCL5, which are important for the regulation of cytokine production, cytokine biosynthetic processes, secretion by cells, chemotaxis, migration, cell motility, the regulation of cell proliferation, and the regulation of cellular differentiation appear to be upregulated (Table S4). A summary of the selected distribution of genes of interest according to the Gene Ontology Classification of Biological Processes is shown in Fig. 3.

Taken together, our analysis of the global gene expression changes revealed that a large number of genes are expressed in different patterns within the SPC subpopulations. First, increased expression of genes associated with the production and secretion of proteins was observed in lineage-negative cells compared to the CD34^+^ or CD133^+^ populations (Table S2). Second, the expression pattern associated with chemotaxis, migration, and cell motility is relatively over-expressed in lineage-negative SPCs compared to CD34^+^ or CD133^+^ cells. Third, genes involved in the regulation of proliferation or differentiation were upregulated in lineage-negative SPCs compared to CD34^+^ or CD133^+^ cells.

### Incubation in stress-related serum-free conditions increases BDNF and NT-3 expressions in NCs and lineage-negative SPCs

**Figure 4:**
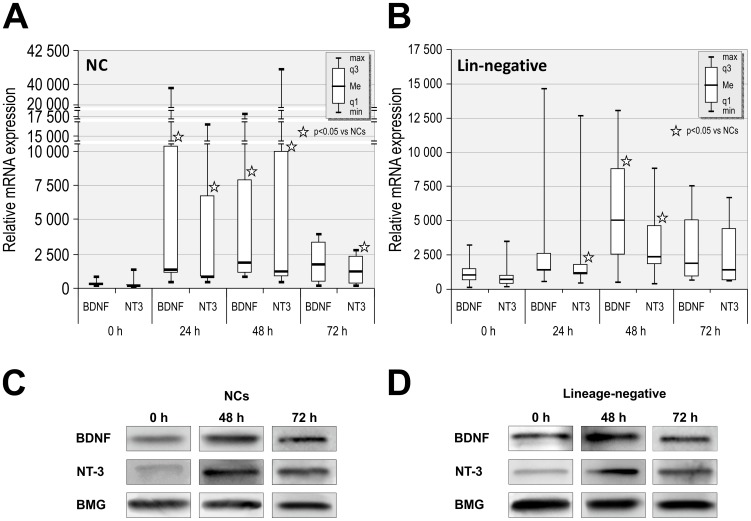
Stress-related, serum-free conditions increases BDNF and NT-3 expression in UCB NCs and lineage-negative cells. (A) QRT-PCR shows changes in mRNA expression of BDNF and NT-3 in NCs after 24, 48, and 72 h in serum-free media. Relative mRNA expression levels are normalized against BMG. Data are shown as median, quartiles, interquartile range, minimum, and maximum for twelve independent experiments. *P<0.05 versus 0 h. (B) QRT-PCR shows changes in the mRNA expression of BDNF and NT-3 in lineage-negative cells after 24, 48, and 72 h in serum-free media. Relative mRNA expression levels are normalized to BMG levels. Data are shown as median, quartiles, interquartile range, minimum, and maximum for six independent experiments. *P<0.05 versus 0 h. (C) Representative western blot analysis of BDNF and NT-3 protein levels in NC lysates. (D) Representative western blot analysis of BDNF and NT-3 protein levels in lineage-negative cell lysates.

Our next aim was to investigate whether stress-related conditions altered the expression of BDNF and NT-3 in UCB-derived lineage-negative SPCs and unsorted NCs. We hypothesized that the production of various soluble factors and NTs by CB-derived SPCs would be increased during stress conditions that are similar to the stress involved in transplantation procedures. BDNF and NT-3 were selected because these NTs are specifically responsible for the maintenance, proliferation, and differentiation of neurons and play an important role in neuroregeneration [25], [26]. Moreover, BDNF has been shown to be particularly effective in combination with NT-3 [27]. Using QRT-PCR, we first investigated the expression levels of BDNF and NT-3 before and after incubation in NCs. We found that BDNF mRNA expression was significantly higher in NCs after 24 and 48 h compared to 0 h controls. However, NT-3 expression levels were elevated after 24, 48 and 72 h incubation (836, 1,182, 1,187; respectively versus 156, p<0.0001) (Fig. 4A).

Next, to evaluate whether stress-related conditions may have similar functional consequences for SPCs, we incubated the lineage-negative cells under serum-free conditions because their gene expression profile suggested they could considerably promote protein synthesis and secretion, cell proliferation, and cell motility. QRT-PCR analysis revealed potent overexpression of BDNF and NT-3 in lineage-negative SPCs after 24 h of incubation under serum-free conditions. Serum-free incubation induced a time-dependent increase in the expression level of NTs. BDNF and NT-3 expression levels were significantly increased in lineage-negative SPCs after 48 h of incubation (2,357 vs. 1,015, p<0.05; and 2,357 vs. 718, p<0.05, respectively; Fig. 4B) compared to 0 h controls. The expression levels of NTs after prolonged incubation were noticeable elevated but did not reach statistical significance. Additional QRT-PCR analysis of BDNF and NT-3 expression levels performed on NCs and lineage-negative cells incubated in medium containing 5% BCS revealed that these expression levels were not significantly elevated at these time points (data not shown).

### Lineage-negative-conditioned medium increases the survival and proliferation of cultured SH-SY5Y cells

**Figure 5:**
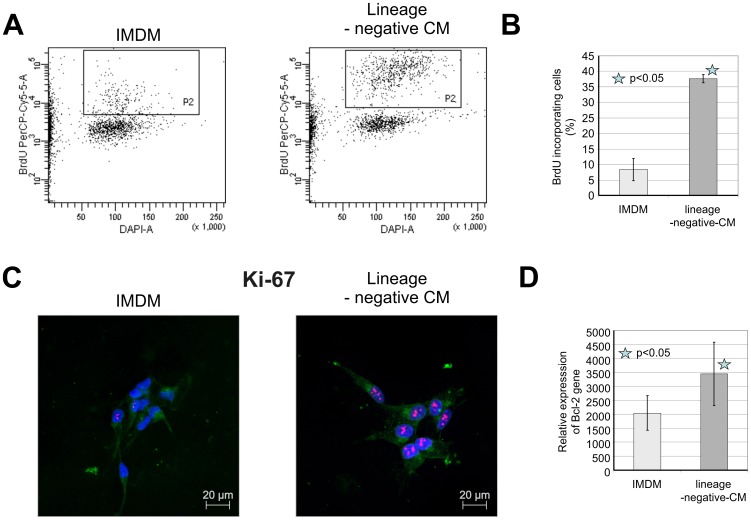
Effects of lineage-negative-conditioned medium on SH-SY5Y cells. Cells were exposed to lineage-negative-CM for 24 h and compared with IMDM-treated cells (control). A. Effects of lineage-negative-CM on BrdU incorporation by SH-SY5Y cells measured by flow cytometry (representative flow cytometry plots). Gate 1, proliferating population indicated by BrdU and DAPI staining (left plot, cells treated with IMDM; right plot, cells treated with lineage-negative-CM). B. Proliferation of SH-SY5Y cells measured by flow cytometry with data expressed as the percentage of proliferating cells. Data are presented as the mean ± S.D. for three independent experiments. *P<0.05 vs. control. C. The expression of the proliferation marker Ki-67 in SH-SY5Y cells was detected by immunofluorescent microscopy. Cells were stained with a monoclonal Texas Red-conjugated anti-Ki-67 antibody. To visualize the cell membrane, cells were stained with an Alexa Fluor 647-conjugated wheat germ agglutinin (WGA). The nuclei were stained with DAPI. Cells were treated for 24 h with IMDM or lineage-negative-CM. All images were acquired by using a LSM700 confocal system (Carl Zeiss, Jena, Germany). D. Expression of Bcl-2 mRNA in SH-SY5Y cells after 24 h treatment of lineage-negative cell conditioned medium detected by QRT-PCR. Data are expressed as a percentage of the control (IMDM-treated cells). Data are presented as the mean ± S.D. for three independent experiments. *P<0.05 vs. control.

Based on these results and given the trophic ability NTs, we investigated whether trophic factors, including NTs produced by lineage-negative cells, could stimulate neuronal cell proliferation and survival. We assessed the effects of trophic factors excreted by lineage-negative cells on the proliferation of the human neural cell line SH-SY5Y. In serum-free conditions, SH-SY5Y cells were subjected to 24 h of lineage-negative conditioned media or IMDM (control), and SH-SY5Y cell proliferation was measured by BrdU incorporation into newly synthesized DNA strands of actively proliferating cells (Fig. 5A). Accordingly, SH-SY5Y cells were cultured for 24 h in serum-free conditions and 25% (v/v) lineage-negative-CM or IMDM was added. After 24 h, BrdU was added for 2 h, and proliferation rates were evaluated using flow cytometry. We observed that after 24 h, lineage-negative-CM increased the percentage of proliferating cells, expressed as cells incorporating BrdU compared with IMDM (Fig. 5B).

To determine whether lineage-negative-CM stimulated neural cell proliferation, SH-SY5Y cells were treated with lineage-negative-CM or IMDM and examined for Ki-67 expression, a proliferation antigen. Ki-67 is present in cells in active phases of the cell cycle (G1, S, G2, and mitosis) and localizes to the nucleus. Ki-67 staining of lineage-negative-CM-treated cells revealed an increase in the number of cells in active cell cycle phases compared with IMDM-treated cells (Fig. 5D). Our results clearly suggest that trophic factors, including NTs expressed and released by lineage-negative SPCs, promote SH-SY5Y cell proliferation.

Along with examining proliferation, we assessed the influence of lineage-negative-CM on apoptosis in SH-SY5Y cells. SH-SY5Y cells were cultured for 24 h in serum-free conditions and 25% (v/v) lineage-negative-CM or IMDM was added. To assess the influence of lineage-negative-CM on apoptosis, we measured mRNA expression of three apoptotic genes, pro-apoptotic Bax, anti-apoptotic Bcl-2, and anti-apoptotic Bcl-xL, in SH-SY5Y cells using qRT-PCR. Apoptosis is a biological process requiring activation of several signaling cascades. It is widely accepted that the mitochondrial pathway, also known as the intrinsic, is one of apoptosis pathways [28]. Bcl-2 and Bax are key proteins in the initiation of programmed cell death and act upstream of caspase-3 and regulate apoptosis induction from different stimuli. We observed that lineage-negative-CM-treated SH-SY5Y cells significantly increased anti-apoptotic Bcl-2 gene expression compared with IMDM-treated cells (Fig. 5B). No expression differences were observed in pro-apoptotic Bax and anti-apoptotic Bcl-xL mRNA levels between lineage-negative-CM- and IMDM-treated cells (data not shown).

Altogether, these results demonstrate that lineage-negative SPCs release neuroregulatory factors that provide a direct beneficial impact on proliferation and survival of neural cells.

## Discussion

In this study, we demonstrate for the first time that human UCB-derived SPC populations constitutively express both NTs as well as their receptors. Concurrently, we determined that NT expression levels in SPCs are higher than in unsorted NCs and that these levels differ between the studied subpopulations. Human UCB is an excellent, readily available source of stem cells for transplantation and that offer the possibility of being used in an autologous and allogenic setting, have genomic stability, have a low risk for cytomegalovirus infections, graft versus host disease, or neoplastic transformation, and lack ethical concerns about their use. Current stem cell transplantation requires specific transplantation consisting of selected SPCs. Accordingly, CD34^+^ populations are widely used not only in routine treatment of hemato-oncologic diseases, but also in experimental stem cell-based therapies in animal models [29]. CD133^+^ and lineage-negative SPCs have been extensively studied in various animal experiments involving stem cell-based approaches as a new strategy for regenerative medicine [11], [30], [31]. In our study, we compared three populations of UCB-derived SPCs: lineage-negative cells, CD34^+^ cells, and CD133^+^ cells. Each of these subpopulations displays unique characteristics in terms of their trophic activity, migration ability, proliferation, and differentiation profiles. Advances in cell isolation techniques have made it possible to obtain highly purified populations of living cells based on surface antigens such as CD34 or CD133. Transplantations of isolated, specific SPCs are safely and widely used. In our study, we used negative selection for lineage-negative SPCs and positive selection for CD34 as well as CD133 cells through immunomagnetic separation. The efficiency achieved in our isolations was similar to that reported by others [32], [33]. However, precise comparison of unique cell populations is problematic due to differences in the preprocessing methodology used. Although it has been reported that positive immunomagnetic selection does not alter hematopoietic SPC functional properties [34], direct antibody ligation is known to potentially mediate cell adhesion molecular activity, including that involving CD34 [35]. Therefore, the use of negative selection protocols avoids the activation of adhesion molecules and offers a real advantage over positive selection. By isolating UCB-derived lineage-negative SPCs, we obtained a heterogeneous population containing both CD34^+^cells, promising immature CD133^+^ cells, and other primitive CD34^−^ stem cells. Previous studies have demonstrated that CD34 can be present intracellularly at the mRNA and/or the protein level without yet being localized to the cell surface in primitive stem cells, and that this population of CD34^-^ cells also contains primitive stem cells [36]. Furthermore, lineage-negative SPCs were abundantly positive for CXCR4 (89.9%), the cognitive SDF-1 receptor, which has previously been reported as essential for hematopoietic SPC homing to and adhesion in the bone marrow as well as for the migration of SPCs from the bloodstream to sites of organ or tissue injury [37]. Our data concerning the expression of surface markers are in concordance those obtained in a previous study by Forraz et al. [38]. Altogether, lineage-negative SPCs may indeed have great potential.

The transplantation of stem cells is a new option for the treatment of neurodegenerative disorders. Growing evidence has documented that UCB-derived cells exert beneficial effects in reducing neuro-functional deficits in experimental animal models of disease. Mononuclear cells (MNCs), as well as other cell populations from human UCB, have been used in animal experiments to regenerate damaged neural tissue with documented therapeutic effects [1]. It has been reported that the administration of UCB-derived CD34^+^ cells into immune compromised mice after middle cerebral artery ligation reduced neurological deficits and induced neuroregeneration partially through the secretion of angiogenic factors [8]. Other studies have determined that systemic application of UCB-derived cells induces neuroregeneration after heatstroke [5], [39]. Neural deficit reduction and diminished effects on lesion volume following middle cerebral artery occlusion has also previously been demonstrated for UCB-derived MNCs [12]. Because previous studies have shown that UCB-derived SPCs possess intrinsic homing ability, migrating to injured neural tissue and actively participating in tissue repair [1], [40], it is strongly suggested that CD34^+^ cells that are able to preferentially home to neural tissue in vitro are particularly involved in these neuroprotective effects. The intracerebroventricular administration of human UCB-derived mesenchymal stem cells in mice after trauma has been shown to stimulate the injured brain and increase the concentration of neurotrophic factors in the lesioned tissue, thereby promoting a microglia/macrophage phenotypic switch and promoting glial scar inhibitory effects that remodel the brain and lead to significant improvement in neurologic outcome [41]. However, the precise therapeutic mechanisms by which UCB-derived cells affect neural tissue regeneration in animal models is complex and remains unclear. Neurotrophic factors secreted by transplanted UCB-derived cells have been strongly suggested to be partially responsible for the amelioration of CNS deficits in animal models after transplantation. NTs may provide neuroprotection by supporting the growth stimulation of neural progenitor cells or by attenuating the apoptotic signaling of chronic inflammation that characterizes neurodegenerative disease. Thus, SPCs could be a source of trophic factors for neural cells afflicted with neurodegenerative disease. In attempting to shed more light on this issue, we assessed the comparative expression of neuroprotective growth factors at the mRNA and protein levels in specific UCB-derived SPC subpopulations.

To date, only selected neurotrophic factors have been investigated in UCB-derived SPCs, and the expression of a wider panel of NTs has not been assessed in these cells. BDNF is one of the most studied and promising growth factors for neuronal regenerative therapy and regulates a number of neuronal functions, including survival, neurogenesis, and the synaptic plasticity of neurons [25]. The neuroprotective effects of BDNF immediately following injury have been documented in studies demonstrating BDNF-induced oligodendrocyte proliferation and the myelination of regenerating axons in a contused adult rat spinal cord, and BDNF-induced axonal regrowth in the presence of a fibroblast graft [42], [43]. The production of BDNF in vitro and in vivo has previously been demonstrated for activated human T cells, B cells, and monocytes from peripheral blood (PB) [44]. Another study has shown that UCB-derived MNCs express the mRNA of several neurotrophic factors at higher levels than adult peripheral blood mononuclear cells (PBMNCs) [2]. Moreover, a significant increase in the levels of BDNF and NT-4 has been found in cultured supernatants of UCB-derived MNCs compared to that of PBMNCs [2]. In this study, freshly isolated lineage-negative, CD34^+^, and CD133^+^ cells effectively expressed BDNF at a comparative level, and their expression was significantly higher than in unsorted NCs. No significant differences in BDNF levels were observed between the three SPC populations studied (lineage-negative, CD34^+^, and CD133^+^). Comparison of the BDNF receptor revealed that all three populations expressed TrkB at the same level, which was higher than that in NCs.

As one of the most potent NTs, NGF exerts a beneficial effect on the survival and maintenance of neuronal functions in the nervous system. One of the first studies concerning the expression of NTs by SPCs revealed that hematopoietic CD34^+^ cells from human UCB express NGF and its receptor TrkA, and that their expression levels are higher than those of either UCB-derived MNCs or PBMNCs [45]. Concordantly, our study shows that NGF expression in UCB-derived CD34^+^ cells is higher than in NCs. Moreover, similar expression levels were seen in CD34^+^ cells and the lineage-negative SPCs, but higher expression levels were observed in CD133^+^ cells. However, the expression of the NGF receptor TrkA was higher in CD34^+^ cells than in lineage-negative SPCs, CD133^+^ cells, and unsorted NCs. NGF, apart from its neurotrophic activities, prevents apoptosis and induces cell proliferation and/or the differentiation of hematopoietic lineages [46]. Our studies revealed that UCB-derived SPCs also strongly and specifically express other NTs, including NT-3, NT-4, and GDNF. Apart from these classical NTs, our three SPC populations also expressed the neuropoietic cytokines CDNF, MANF, and PEDF. These cytokines demonstrated neuroprotective attributes, as did VEGF, which has angiogenic and neurotrophic activities. All three of these SPC populations expressed neurotrophic factors at higher levels than unsorted NCs. CDNF, a neurotrophin that protects dopaminergic neurons, was expressed at a higher level in CD34^+^ and CD133^+^ cells than in lineage-negative SPCs; a similar result was seen for the PEDF cytokine, which exerts neuroprotective and anti-angiogenic effects. Our data elucidates a unique profile for each UCB-derived SPC population that could be selected based on the specific application required. Our results suggest that SPC populations may effectively produce NTs. Secreted factors may reasonably represent the humoral background of UCB-derived SPCs neuroprotection. In addition, the angiopoietic growth factor VEGF might play a role in neural regeneration by supporting angiogenesis and promoting neuroprotection [47].

The use of SPCs for neurotrophic therapy constitutes a promising approach for the treatment of neurodegenerative diseases. Ideally, such cells should exhibit two key properties: (i) a high level of neurotrophic factor synthesis in vitro, which would allow for the secretion of trophic factors at the transplantation site, and (ii) chemotaxis, which would facilitate the ability to reach degenerative processes in specific tissues. To further characterize UCB-derived SPC populations in this context, we compared global gene expression patterns between freshly isolated lineage-negative cells, CD34^+^, and CD133^+^ cells. The gene expression profiles of human CD34^+^ and CD133^+^ cells have previously been reported [48], [49]. A study comparing the genome-wide gene expression profiles of UCB-derived CD34^+^ and CD133^+^ cells indicated the primitive nature of CD133^+^ cells [50]. In our study, we were interested in biological processes concerning biosynthetic processing, cytokine production, secretion by cells, chemotaxis, migration, and proliferative capacities. Comparison of the bioinformatic analyses of the complex gene datasets in lineage-negative SPCs and CD34^+^ cells identified that, among others, the most upregulated genes were involved in the regulation of cytokine production, secretion by cells, exocytosis, the production of tumor necrosis factor superfamily cytokines, chemotaxis, cell migration, cell motility, the regulation of chemotaxis, and the positive regulation of cellular differentiation. Lineage-negative SPCs, when compared to CD133^+^ cells, overexpressed several genes that are important for the regulation of cytokine production, cytokine biosynthetic processes, secretion by cells, chemotaxis, migration, cell motility, and the regulation of cellular proliferation and differentiation. Our comparison of the gene expression profiles of lineage-negative, CD34^+^, and CD133^+^ SPCs allowed us to conclude that genes associated with the production and secretion of proteins as well as genes responsible for chemotaxis, migration, and cell motility were overexpressed in lineage-negative SPCs compared to both CD34^+^ and CD133^+^ cells. The results generated in this microarray analysis may elucidate new perspectives when studying the functional differences between various SPC populations. Altogether, gene expression profiling of lineage-negative SPCs appears to favor the idea that these cells have trophic activities in damaged tissue. These data have provided new insights into the underlying complexity of SPC biology and are expected to offer novel clues in improving our understanding and application of SPCs in the clinic.

Neurotrophin deficiency plays a crucial role in the pathological mechanisms involved in neurodegenerative processes. In the CNS, BDNF has been found to be an important neurotrophin for cholinergic neurons (which are affected in Alzheimer's disease) and for motor neurons that degenerate in amyotrophic lateral sclerosis (ALS) [51], [52]. BDNF has also been described to play a neuroprotective role for dopaminergic neurons that are depleted in the substantia nigra in Parkinson's disease [53]. NTs are also used in experimental treatments of other selected neurodegenerative diseases. In clinical trials, GDNF has been used in the treatment of Parkinson's disease, BDNF in the treatment of ALS, and NGF as a therapeutic agent in Alzheimer's disease. However, delivering these proteins to the appropriate place in the human brain has encountered obstacles, including their unfavorable pharmacokinetic profile, short serum half-life, and high manufacturing costs. In addition, NTs are relatively large, polar molecules that cannot readily pass through the blood-brain barrier and therefore need to be administered directly into the CNS. Once at the site of injury, SPCs can secrete NTs that may lead to improvement in the cellular activities of endogenous cells and factors that are released during neuroregeneration.

Because BDNF and NT-3 play important roles as neuro-regenerative factors, we decided to compare the changes in the expression levels of these NTs in UCB-derived cells under stress-related conditions. NT-3 mediates almost all neuron survival and differentiation-promoting activities in the central and peripheral nervous systems. NT-3 diminishes neuronal loss, supports axonal elongation, and promotes angiogenesis and neurogenesis. BDNF and NT-3 produced by SPCs play crucial roles in neuronal survival and in the regenerative process that follows peripheral nerve injury [54], [55]. The data presented here also demonstrated that stress affects the expression and production of the neurotrophic factors BDNF and NT3 in UCB-derived NCs and SPCs. In this study, we have hypothesized that SPCs respond to stress by increasing the expression of various genes and that this may be a favorable therapeutic modality to enhance their trophic activities to promote neuronal growth and neuroprotection during stem cell-based therapy. Here, we have shown that the production of the regenerative NTs BDNF and NT-3 by NCs, as well as lineage-negative SPCs, was robustly increased under stress-related serum-free conditions. Similar effects were previously observed in hypoxic conditions in bone marrow mesenchymal stem cells (BMSCs). Hypoxic preconditioning of BMSCs not only enhanced their survival but also reinforced the regenerative properties of these cells, promoting their regenerative capability and therapeutic potential after transplantation [21]. Similarly, another recent study has revealed that under hypoxic conditions, UCB-derived MSCs adapted their cytokine expression by upregulating several growth factors [56]. Our study demonstrates that the production of various neuropeptides (including NTs) by UCB-derived SPCs can be increased by exposure of these cells to stress conditions. The isolation process and subsequent therapeutic administration of cells into the cerebrospinal fluid (CSF), which contains a relatively low level of plasma proteins, as well as into eye vitreous in age-related macular degeneration, induces stress in these cells. Hence, we suggest that the neural microenvironment of transplanted cells might accelerate the therapeutic potential of UCB-derived SPCs by increasing the production of neurotrophic factors, thereby improving the regeneration of neural tissue. Thus, important insights into the activity of SPCs in regenerative processes during healing after injury have resulted from this study by clarifying the types and levels of neuroprotective NTs produced by these cells under steady-state and stress-related conditions.

Based on these results and given the ability of NTs to promote neural cell survival and proliferation, we investigated the influence of lineage-negative-conditioned medium on the proliferation and survival of neural cells in vitro. Our data showed that lineage-negative-CM favors proliferation of human SH-SY5Y cells and revealed anti-apoptotic properties. In other studies, different SPC-conditioned media exerted beneficial effects on treated cells [57]–[60]. To optimize potential effects of soluble factors secreted by lineage-negative SPCs, we decided to deliver concentrated conditioned medium from these cells and assess the influence on SH-SY5Y cell proliferation and survival. This method has been previously documented as effective in experimental settings [57], [58]. Because serum is a source of nutrients and other ill-defined factors, we excluded it from the medium in our experiments. Bakondi et al. found that conditioned medium from immunomagnetically isolated BM-derived CD133^+^ cells provided significant protection against growth factor/nutrient withdrawal-induced cell death compared with serum-free medium in neural progenitor cells [57]. They also demonstrated that intra-arterial infusion of concentrated CD133^+^-conditioned medium provided protection against stroke in immunodeficient mice with cerebral ischemia. Cantinieaux at al. demonstrated that in vitro BM-derived MSC-conditioned medium protected against neuronal apoptosis [58]. For umbilical cord cells, conditioned medium from mesenchymal progenitors present in umbilical cord and Wharton's jelly has been found to increase viability and proliferation of glial and primary hippocampal cells in culture [59]. Yang et al. revealed that soluble factors secreted by endothelial progenitor cells exerted strong cyto-protective effects on differentiated endothelium by modulating intracellular antioxidant defensive mechanisms and pro-survival signals [60]. Considering the beneficial effects of SPC-secreted trophic factors, it has been hypothesized that secreted factors in MSC-conditioned medium could be used to prime tissue-specific SPCs and improve graft success after transplantation of these cells. In a very recent study, Iso et al. demonstrated that short-term priming of cardiac SPCs with MSC-conditioned medium pre-transplantation into sub-epicardial tissue markedly improved engraftment [61].

Taken together, these findings demonstrate for the first time that UCB-derived SPCs strongly and specifically express the classical NTs BDNF, NGF, NT-3, NT-4, and GDNF, as well as the novel neurotrophic cytokines CDNF, MANF, PEDF, and VEGF. These cells also possess the neurotrophin receptors p75NTR, TrkA, TrkB, TrkC, GRFα1, and GFRα2 at the transcriptional and post-transcriptional levels. We found that NT expression in SPC populations was higher in SPCs than in unsorted NCs. Immunomagnetic cell separation enables the rapid and gentle sorting of SPCs to prepare specific cell types for use in research and clinical applications. We also demonstrated that secreted factors in conditioned medium from lineage-negative SPCs support neuronal proliferation and survival in vitro. Because UCB-derived SPCs express and produce NTs, they represent good candidates for restoring damaged areas of neural tissue, where regeneration and neuroprotection depend on the appropriate accessibility of these factors. Neurotrophic factors are produced in different patterns among the various SPC populations, suggesting that one of these populations might be more effective for regeneration in specific degenerative processes. Our data suggests that UCB-derived SPC populations may play an important role in the regeneration of damaged neural tissue and its protection by promoting neurogenesis and angiogenesis as well as decreasing the apoptosis of neural cells. The application of UCB-derived SPCs may provide therapeutic benefits via their humoral activity by releasing soluble trophic factors. Knowing the expression profiles of the various SPC subpopulations is critical to understanding the trophic effects of these cells within the neurogenic niche where they are recruited or transplanted; we hope that this study will help to determine potential clinical applications for these cells. The stimulation of endogenous cells using exogenous stem cells for supporting trophic factors is a promising attempt at tissue repair. Functional increases in NT production that are associated with stress-related conditions in SPC populations in vitro may have important clinical implications. Knowledge of the mechanisms governing the characterization and humoral activity of various subsets of SPCs originating from different sources may give rise to new therapeutic strategies that are more effective for treating neurodegenerative disorders.

## Supporting Information

Table S1 **The ten upregulated genes with the largest change in expression for the lineage-negative SPCs compared to the CD34^+^.**
(DOC)

Table S2 **The ten upregulated genes with the largest change in expression for the lineage-negative SPCs compared to the CD133^+^.**
(DOC)

Table S3 **Selected genes and pathways of interest from our significant gene list in lineage-negative cells that are overexpressed compared to CD34^+^ cells.**
(DOC)

Table S4 **Selected genes and pathways of interest from our significant gene list in lineage-negative cells that are overexpressed compared to CD133^+^ cells.**
(DOC)
