# Supplementary material for: Humoral Activity of Cord Blood-Derived Stem/Progenitor Cells: Implications for Stem Cell-Based Adjuvant Therapy of Neurodegenerative Disorders
Source: PLoS One. 2013 Dec 31;8(12):e83833. doi: 10.1371/journal.pone.0083833 (PMC3877125; doi:10.1371/journal.pone.0083833)
Supplement: Table S1 — The ten upregulated genes with the largest change in expression for the lineage-negative SPCs compared to the CD34+. (DOC) [file pone.0083833.s001.doc]

**Table S1. The ten upregulated genes with the largest change in expression for the lineage-negative SPCs compared to the CD34^+^**.

| ProbeID | GeneSymbol | log_2_(FC) | GeneName | EntrezGeneID |
| --- | --- | --- | --- | --- |
| 8007931 | ITGB3 | 4.288 | integrin, beta 3 (platelet glycoprotein IIIa, antigen CD61) | 3690 |
| 8024038 | AZU1 | 4.072 | azurocidin 1 | 566 |
| 8008723 | EPX | 4.01 | eosinophil peroxidase | 8288 |
| 8024056 | ELANE | 3.921 | elastase, neutrophil expressed | 1991 |
| 8100971 | PPBP | 3.562 | pro-platelet basic protein (chemokine (C-X-C motif) ligand 7) | 5473 |
| 7973110 | RNASE2 | 3.539 | ribonuclease, RNase A family, 2 (liver, eosinophil-derived neurotoxin) | 6036 |
| 8137670 | PDGFA | 3.471 | platelet-derived growth factor alpha polypeptide | 5154 |
| 8016044 | ITGA2B | 3.422 | integrin, alpha 2b (platelet glycoprotein IIb of IIb/IIIa complex, antigen CD41) | 3674 |
| 7922200 | SELP | 3.378 | selectin P (granule membrane protein 140kDa, antigen CD62) | 6403 |
| 8085062 | IL5RA | 3.136 | interleukin 5 receptor, alpha | 3568 |
