# Supplementary material for: Humoral Activity of Cord Blood-Derived Stem/Progenitor Cells: Implications for Stem Cell-Based Adjuvant Therapy of Neurodegenerative Disorders
Source: PLoS One. 2013 Dec 31;8(12):e83833. doi: 10.1371/journal.pone.0083833 (PMC3877125; doi:10.1371/journal.pone.0083833)
Supplement: Table S2 — The ten upregulated genes with the largest change in expression for the lineage-negative SPCs compared to the CD133+. (DOC) [file pone.0083833.s002.doc]

**Table S2. The ten upregulated genes with the largest change in expression for the lineage-negative SPCs compared to the CD133^+^.**

| ProbeID | GeneSymbol | log_2_(FC) | GeneName | EntrezGeneID |
| --- | --- | --- | --- | --- |
| 8024056 | ELANE | 5.053 | elastase, neutrophil expressed | 1991 |
| 8008723 | EPX | 4.759 | eosinophil peroxidase | 8288 |
| 8024038 | AZU1 | 4.42 | azurocidin 1 | 566 |
| 7973110 | RNASE2 | 4.144 | ribonuclease, RNase A family, 2 (liver, eosinophil-derived neurotoxin) | 6036 |
| 8085062 | IL5RA | 4.031 | interleukin 5 receptor, alpha | 3568 |
| 8016044 | ITGA2B | 4.003 | integrin, alpha 2b (platelet glycoprotein IIb of IIb/IIIa complex, antigen CD41) | 3674 |
| 8062444 | BPI | 3.566 | bactericidal/permeability-increasing protein | 671 |
| 8137670 | PDGFA | 3.289 | platelet-derived growth factor alpha polypeptide | 5154 |
| 8100971 | PPBP | 3.237 | pro-platelet basic protein (chemokine (C-X-C motif) ligand 7) | 5473 |
| 8002249 | SMPD3 | 3.231 | sphingomyelin phosphodiesterase 3, neutral membrane (neutral sphingomyelinase II) | 55512 |
