# Supplementary material for: Humoral Activity of Cord Blood-Derived Stem/Progenitor Cells: Implications for Stem Cell-Based Adjuvant Therapy of Neurodegenerative Disorders
Source: PLoS One. 2013 Dec 31;8(12):e83833. doi: 10.1371/journal.pone.0083833 (PMC3877125; doi:10.1371/journal.pone.0083833)
Supplement: Table S3 — Selected genes and pathways of interest from our significant gene list in lineage-negative cells that are overexpressed compared to CD34+ cells. (DOC) [file pone.0083833.s003.doc]

**Table S3. Selected genes and pathways of interest from our significant gene list in lineage-negative cells that are overexpressed compared to CD34^+^ cells.**

| REGULATION OF CYTOKINE PRODUCTION | | | | |
| --- | --- | --- | --- | --- |
| GeneSymbol | GeneName | ProbeID | EntrezGeneID | log_2_(FC) |
| AZU1 | azurocidin 1 | 8024038 | 566 | 4.072 |
| EPX | eosinophil peroxidase | 8008723 | 8288 | 4.01 |
| ELANE | elastase, neutrophil expressed | 8024056 | 1991 | 3.921 |
| IL5RA | interleukin 5 receptor, alpha | 8085062 | 3568 | 3.136 |
| THBS1 | thrombospondin 1 | 7982597 | 7057 | 3.079 |
| PRG3 | proteoglycan 3 | 7948213 | 10394 | 2.637 |
| SEMA7A | semaphorin 7A, GPI membrane anchor (John Milton Hagen blood group) | 7990345 | 8482 | 2.524 |
| BPI | bactericidal/permeability-increasing protein | 8062444 | 671 | 2.512 |
| IL1RL1 | interleukin 1 receptor-like 1 | 8044021 | 9173 | 2.417 |
| TRIM16 | tripartite motif containing 16 | 8012953 | 10626 | 2.087 |
| EXOCYTOSIS | | | | |
| GeneSymbol | GeneName | ProbeID | EntrezGeneID | log_2_(FC) |
| ITGB3 | integrin, beta 3 (platelet glycoprotein IIIa, antigen CD61) | 8007931 | 3690 | 4.288 |
| PPBP | pro-platelet basic protein (chemokine (C-X-C motif) ligand 7) | 8100971 | 5473 | 3.562 |
| PDGFA | platelet-derived growth factor alpha polypeptide | 8137670 | 5154 | 3.471 |
| ITGA2B | integrin, alpha 2b (platelet glycoprotein IIb of IIb/IIIa complex, antigen CD41) | 8016044 | 3674 | 3.422 |
| SELP | selectin P (granule membrane protein 140kDa, antigen CD62) | 7922200 | 6403 | 3.378 |
| RAB27B | RAB27B, member RAS oncogene family | 8021301 | 5874 | 3.155 |
| THBS1 | thrombospondin 1 | 7982597 | 7057 | 3.079 |
| CCL5 | chemokine (C-C motif) ligand 5 | 8014316 | 6352 | 2.732 |
| ANK1 | ankyrin 1, erythrocytic | 8150439 | 286 | 2.393 |
| F13A1 | coagulation factor XIII, A1 polypeptide | 8123744 | 2162 | 2.342 |
| CHEMOTAXIS | | | | |
| GeneSymbol | GeneName | ProbeID | EntrezGeneID | log_2_(FC) |
| ITGB3 | integrin, beta 3 (platelet glycoprotein IIIa, antigen CD61) | 8007931 | 3690 | 4.288 |
| AZU1 | azurocidin 1 | 8024038 | 566 | 4.072 |
| ELANE | elastase, neutrophil expressed | 8024056 | 1991 | 3.921 |
| PPBP | pro-platelet basic protein (chemokine (C-X-C motif) ligand 7) | 8100971 | 5473 | 3.562 |
| RNASE2 | ribonuclease, RNase A family, 2 (liver, eosinophil-derived neurotoxin) | 7973110 | 6036 | 3.539 |
| PDGFA | platelet-derived growth factor alpha polypeptide | 8137670 | 5154 | 3.471 |
| ITGA2B | integrin, alpha 2b (platelet glycoprotein IIb of IIb/IIIa complex, antigen CD41) | 8016044 | 3674 | 3.422 |
| CMTM5 | CKLF-like MARVEL transmembrane domain containing 5 | 7973403 | 116173 | 3.34 |
| THBS1 | thrombospondin 1 | 7982597 | 7057 | 3.079 |
| CCL5 | chemokine (C-C motif) ligand 5 | 8014316 | 6352 | 2.732 |
| CELL MIGRATION | | | | |
| GeneSymbol | GeneName | ProbeID | EntrezGeneID | log_2_(FC) |
| ITGB3 | integrin, beta 3 (platelet glycoprotein IIIa, antigen CD61) | 8007931 | 3690 | 4.288 |
| AZU1 | azurocidin 1 | 8024038 | 566 | 4.072 |
| EPX | eosinophil peroxidase | 8008723 | 8288 | 4.01 |
| PDGFA | platelet-derived growth factor alpha polypeptide | 8137670 | 5154 | 3.471 |
| ALOX12 | arachidonate 12-lipoxygenase | 8004221 | 239 | 3.413 |
| SELP | selectin P (granule membrane protein 140kDa, antigen CD62) | 7922200 | 6403 | 3.378 |
| THBS1 | thrombospondin 1 | 7982597 | 7057 | 3.079 |
| CCL5 | chemokine (C-C motif) ligand 5 | 8014316 | 6352 | 2.732 |
| CSF1 | colony stimulating factor 1 (macrophage) | 7903786 | 1435 | 2.634 |
| GP6 | glycoprotein VI (platelet) | 8039294 | 51206 | 2.447 |
| SECRETION BY CELL | | | | |
| GeneSymbol | GeneName | ProbeID | EntrezGeneID | log_2_(FC) |
| ITGB3 | integrin, beta 3 (platelet glycoprotein IIIa, antigen CD61) | 8007931 | 3690 | 4.288 |
| PPBP | pro-platelet basic protein (chemokine (C-X-C motif) ligand 7) | 8100971 | 5473 | 3.562 |
| PDGFA | platelet-derived growth factor alpha polypeptide | 8137670 | 5154 | 3.471 |
| ITGA2B | integrin, alpha 2b (platelet glycoprotein IIb of IIb/IIIa complex, antigen CD41) | 8016044 | 3674 | 3.422 |
| SELP | selectin P (granule membrane protein 140kDa, antigen CD62) | 7922200 | 6403 | 3.378 |
| SMPD3 | sphingomyelin phosphodiesterase 3, neutral membrane (neutral sphingomyelinase II) | 8002249 | 55512 | 3.22 |
| RAB27B | RAB27B, member RAS oncogene family | 8021301 | 5874 | 3.155 |
| THBS1 | thrombospondin 1 | 7982597 | 7057 | 3.079 |
| PRKAR2B | protein kinase, cAMP-dependent, regulatory, type II, beta | 8135378 | 5577 | 2.746 |
| CCL5 | chemokine (C-C motif) ligand 5 | 8014316 | 6352 | 2.732 |
| POSITIVE REGULATION OF CELL DIFFERENTIATION | | | | |
| GeneSymbol | GeneName | ProbeID | EntrezGeneID | log_2_(FC) |
| ALOX12 | arachidonate 12-lipoxygenase | 8004221 | 239 | 3.413 |
| CSF1 | colony stimulating factor 1 (macrophage) | 7903786 | 1435 | 2.634 |
| PDE5A | phosphodiesterase 5A, cGMP-specific | 8102532 | 8654 | 2.53 |
| SEMA7A | semaphorin 7A, GPI membrane anchor (John Milton Hagen blood group) | 7990345 | 8482 | 2.524 |
| GATA1 | GATA binding protein 1 (globin transcription factor 1) | 8167360 | 2623 | 2.45 |
| CAMK1 | calcium/calmodulin-dependent protein kinase I | 8085206 | 8536 | 2.294 |
| DAB2 | disabled homolog 2, mitogen-responsive phosphoprotein (Drosophila) | 8111772 | 1601 | 2.219 |
| BAMBI | BMP and activin membrane-bound inhibitor homolog (Xenopus laevis) | 7926875 | 25805 | 2.168 |
| TRIM16 | tripartite motif containing 16 | 8012953 | 10626 | 2.087 |
| PF4 | platelet factor 4 | 8100966 | 5196 | 2.078 |
| POSITIVE REGULATION OF BIOLOGICAL PROCESS | | | | |
| GeneSymbol | GeneName | ProbeID | EntrezGeneID | log_2_(FC) |
| ITGB3 | integrin, beta 3 (platelet glycoprotein IIIa, antigen CD61) | 8007931 | 3690 | 4.288 |
| AZU1 | azurocidin 1 | 8024038 | 566 | 4.072 |
| EPX | eosinophil peroxidase | 8008723 | 8288 | 4.01 |
| CTSG | cathepsin G | 7978351 | 1511 | 3.979 |
| ELANE | elastase, neutrophil expressed | 8024056 | 1991 | 3.921 |
| CTDNEP1 | CTD nuclear envelope phosphatase 1 | 7893729 | 23399 | 3.657 |
| PPBP | pro-platelet basic protein (chemokine (C-X-C motif) ligand 7) | 8100971 | 5473 | 3.562 |
| PDGFA | platelet-derived growth factor alpha polypeptide | 8137670 | 5154 | 3.471 |
| ALOX12 | arachidonate 12-lipoxygenase | 8004221 | 239 | 3.413 |
| PRTN3 | proteinase 3 | 8024048 | 5657 | 3.41 |
