# Supplementary material for: Humoral Activity of Cord Blood-Derived Stem/Progenitor Cells: Implications for Stem Cell-Based Adjuvant Therapy of Neurodegenerative Disorders
Source: PLoS One. 2013 Dec 31;8(12):e83833. doi: 10.1371/journal.pone.0083833 (PMC3877125; doi:10.1371/journal.pone.0083833)
Supplement: Table S4 — Selected genes and pathways of interest from our significant gene list in lineage-negative cells that are overexpressed compared to CD133+ cells. (DOC) [file pone.0083833.s004.doc]

**Table S4. Selected genes and pathways of interest from our significant gene list in lineage-negative cells that are overexpressed compared to CD133^+^ cells.**

| CYTOKINE BIOSYNTHETIC PROCESS | | | | |
| --- | --- | --- | --- | --- |
| GeneSymbol | GeneName | ProbeID | EntrezGeneID | log_2_(FC) |
| ELANE | elastase, neutrophil expressed | 8024056 | 1991 | 5.053 |
| AZU1 | azurocidin 1 | 8024038 | 566 | 4.42 |
| PRG3 | proteoglycan 3 | 7948213 | 10394 | 2.826 |
| CEBPE | CCAAT/enhancer binding protein (C/EBP), epsilon | 7977928 | 1053 | 2.014 |
| REGULATION OF CYTOKINE PRODUCTION | | | | |
| GeneSymbol | GeneName | ProbeID | EntrezGeneID | log_2_(FC) |
| ELANE | elastase, neutrophil expressed | 8024056 | 1991 | 5.053 |
| EPX | eosinophil peroxidase | 8008723 | 8288 | 4.759 |
| AZU1 | azurocidin 1 | 8024038 | 566 | 4.42 |
| IL5RA | interleukin 5 receptor, alpha | 8085062 | 3568 | 4.031 |
| BPI | bactericidal/permeability-increasing protein | 8062444 | 671 | 3.566 |
| PRG3 | proteoglycan 3 | 7948213 | 10394 | 2.826 |
| IL1RL1 | interleukin 1 receptor-like 1 | 8044021 | 9173 | 2.642 |
| RGCC | regulator of cell cycle | 7968789 | 28984 | 2.518 |
| PF4 | platelet factor 4 | 8100966 | 5196 | 2.373 |
| IDO1 | indoleamine 2,3-dioxygenase 1 | 8146092 | 3620 | 2.246 |
| CHEMOTAXIS | | | | |
| GeneSymbol | GeneName | ProbeID | EntrezGeneID | log_2_(FC) |
| ELANE | elastase, neutrophil expressed | 8024056 | 1991 | 5.053 |
| ITGB3 | integrin, beta 3 (platelet glycoprotein IIIa, antigen CD61) | 8007931 | 3690 | 4.681 |
| AZU1 | azurocidin 1 | 8024038 | 566 | 4.42 |
| RNASE2 | ribonuclease, RNase A family, 2 (liver, eosinophil-derived neurotoxin) | 7973110 | 6036 | 4.144 |
| ITGA2B | integrin, alpha 2b (platelet glycoprotein IIb of IIb/IIIa complex, antigen CD41) | 8016044 | 3674 | 4.003 |
| PDGFA | platelet-derived growth factor alpha polypeptide | 8137670 | 5154 | 3.289 |
| PPBP | pro-platelet basic protein (chemokine (C-X-C motif) ligand 7) | 8100971 | 5473 | 3.237 |
| CMTM5 | CKLF-like MARVEL transmembrane domain containing 5 | 7973403 | 116173 | 3.209 |
| ANK1 | ankyrin 1, erythrocytic | 8150439 | 286 | 2.922 |
| CCR3 | chemokine (C-C motif) receptor 3 | 8079383 | 1232 | 2.56 |
| CELL MIGRATION | | | | |
| GeneSymbol | GeneName | ProbeID | EntrezGeneID | log_2_(FC) |
| EPX | eosinophil peroxidase | 8008723 | 8288 | 4.759 |
| ITGB3 | integrin, beta 3 (platelet glycoprotein IIIa, antigen CD61) | 8007931 | 3690 | 4.681 |
| AZU1 | azurocidin 1 | 8024038 | 566 | 4.42 |
| SELP | selectin P (granule membrane protein 140kDa, antigen CD62) | 7922200 | 6403 | 3.726 |
| PDGFA | platelet-derived growth factor alpha polypeptide | 8137670 | 5154 | 3.289 |
| ALOX12 | arachidonate 12-lipoxygenase | 8004221 | 239 | 3.054 |
| ADORA3 | adenosine A3 receptor | 7918533 | 140 | 3.031 |
| GP6 | glycoprotein VI (platelet) | 8039294 | 51206 | 2.781 |
| TACSTD2 | tumor-associated calcium signal transducer 2 | 7916584 | 4070 | 2.521 |
| CSF1 | colony stimulating factor 1 (macrophage) | 7903786 | 1435 | 2.518 |
| SECRETION | | | | |
| GeneSymbol | GeneName | ProbeID | EntrezGeneID | log_2_(FC) |
| ITGB3 | integrin, beta 3 (platelet glycoprotein IIIa, antigen CD61) | 8007931 | 3690 | 4.681 |
| ITGA2B | integrin, alpha 2b (platelet glycoprotein IIb of IIb/IIIa complex, antigen CD41) | 8016044 | 3674 | 4.003 |
| SELP | selectin P (granule membrane protein 140kDa, antigen CD62) | 7922200 | 6403 | 3.726 |
| PDGFA | platelet-derived growth factor alpha polypeptide | 8137670 | 5154 | 3.289 |
| PPBP | pro-platelet basic protein (chemokine (C-X-C motif) ligand 7) | 8100971 | 5473 | 3.237 |
| SMPD3 | sphingomyelin phosphodiesterase 3, neutral membrane (neutral sphingomyelinase II) | 8002249 | 55512 | 3.231 |
| ADORA3 | adenosine A3 receptor | 7918533 | 140 | 3.031 |
| ANK1 | ankyrin 1, erythrocytic | 8150439 | 286 | 2.922 |
| CA2 | carbonic anhydrase II | 8147132 | 760 | 2.755 |
| P2RY2 | purinergic receptor P2Y, G-protein coupled, 2 | 7942400 | 5029 | 2.732 |
| POSITIVE REGULATION OF SECRETION | | | | |
| GeneSymbol | GeneName | ProbeID | EntrezGeneID | log_2_(FC) |
| ADORA3 | adenosine A3 receptor | 7918533 | 140 | 3.031 |
| P2RY2 | purinergic receptor P2Y, G-protein coupled, 2 | 7942400 | 5029 | 2.732 |
| IL1RL1 | interleukin 1 receptor-like 1 | 8044021 | 9173 | 2.642 |
| RGCC | regulator of cell cycle | 7968789 | 28984 | 2.518 |
| RAB27B | RAB27B, member RAS oncogene family | 8021301 | 5874 | 2.484 |
| DOC2B | Double C2-like domain-containing protein beta | 8010901 | 8447 | 2.331 |
| BMP6 | bone morphogenetic protein 6 | 8116818 | 654 | 2.165 |
| CLEC5A | C-type lectin domain family 5, member A | 8143471 | 23601 | 2.133 |
| ADORA3 | adenosine A3 receptor | 7918533 | 140 | 3.031 |
| P2RY2 | purinergic receptor P2Y, G-protein coupled, 2 | 7942400 | 5029 | 2.732 |
| CELL MOTILITY | | | | |
| GeneSymbol | GeneName | ProbeID | EntrezGeneID | log_2_(FC) |
| EPX | eosinophil peroxidase | 8008723 | 8288 | 4.759 |
| ITGB3 | integrin, beta 3 (platelet glycoprotein IIIa, antigen CD61) | 8007931 | 3690 | 4.681 |
| AZU1 | azurocidin 1 | 8024038 | 566 | 4.42 |
| SELP | selectin P (granule membrane protein 140kDa, antigen CD62) | 7922200 | 6403 | 3.726 |
| PDGFA | platelet-derived growth factor alpha polypeptide | 8137670 | 5154 | 3.289 |
| ALOX12 | arachidonate 12-lipoxygenase | 8004221 | 239 | 3.054 |
| ADORA3 | adenosine A3 receptor | 7918533 | 140 | 3.031 |
| GP6 | glycoprotein VI (platelet) | 8039294 | 51206 | 2.781 |
| TACSTD2 | tumor-associated calcium signal transducer 2 | 7916584 | 4070 | 2.521 |
| CSF1 | colony stimulating factor 1 (macrophage) | 7903786 | 1435 | 2.518 |
| REGULATION OF LOCOMOTION | | | | |
| GeneSymbol | GeneName | ProbeID | EntrezGeneID | log_2_(FC) |
| ELANE | elastase, neutrophil expressed | 8024056 | 1991 | 5.053 |
| ITGB3 | integrin, beta 3 (platelet glycoprotein IIIa, antigen CD61) | 8007931 | 3690 | 4.681 |
| AZU1 | azurocidin 1 | 8024038 | 566 | 4.42 |
| SELP | selectin P (granule membrane protein 140kDa, antigen CD62) | 7922200 | 6403 | 3.726 |
| PDGFA | platelet-derived growth factor alpha polypeptide | 8137670 | 5154 | 3.289 |
| ALOX12 | arachidonate 12-lipoxygenase | 8004221 | 239 | 3.054 |
| ADORA3 | adenosine A3 receptor | 7918533 | 140 | 3.031 |
| TACSTD2 | tumor-associated calcium signal transducer 2 | 7916584 | 4070 | 2.521 |
| CSF1 | colony stimulating factor 1 (macrophage) | 7903786 | 1435 | 2.518 |
| RGCC | regulator of cell cycle | 7968789 | 28984 | 2.518 |
| REGULATION OF CELL PROLIFERATION | | | | |
| GeneSymbol | GeneName | ProbeID | EntrezGeneID | log_2_(FC) |
| ELANE | elastase, neutrophil expressed | 8024056 | 1991 | 5.053 |
| ITGB3 | integrin, beta 3 (platelet glycoprotein IIIa, antigen CD61) | 8007931 | 3690 | 4.681 |
| PRTN3 | proteinase 3 | 8024048 | 5657 | 4.142 |
| PDGFA | platelet-derived growth factor alpha polypeptide | 8137670 | 5154 | 3.289 |
| ALOX12 | arachidonate 12-lipoxygenase | 8004221 | 239 | 3.054 |
| GATA1 | GATA binding protein 1 (globin transcription factor 1) | 8167360 | 2623 | 2.918 |
| PDE5A | phosphodiesterase 5A, cGMP-specific | 8102532 | 8654 | 2.695 |
| TACSTD2 | tumor-associated calcium signal transducer 2 | 7916584 | 4070 | 2.521 |
| CSF1 | colony stimulating factor 1 (macrophage) | 7903786 | 1435 | 2.518 |
| RGCC | regulator of cell cycle | 7968789 | 28984 | 2.518 |
| POSITIVE REGULATION OF BIOLOGICAL PROCESS | | | | |
| GeneSymbol | GeneName | ProbeID | EntrezGeneID | log_2_(FC) |
| CTSG | cathepsin G | 7978351 | 1511 | 5.403 |
| ELANE | elastase, neutrophil expressed | 8024056 | 1991 | 5.053 |
| EPX | eosinophil peroxidase | 8008723 | 8288 | 4.759 |
| ITGB3 | integrin, beta 3 (platelet glycoprotein IIIa, antigen CD61) | 8007931 | 3690 | 4.681 |
| AZU1 | azurocidin 1 | 8024038 | 566 | 4.42 |
| PRTN3 | proteinase 3 | 8024048 | 5657 | 4.142 |
| SELP | selectin P (granule membrane protein 140kDa, antigen CD62) | 7922200 | 6403 | 3.726 |
| PDGFA | platelet-derived growth factor alpha polypeptide | 8137670 | 5154 | 3.289 |
| PPBP | pro-platelet basic protein (chemokine (C-X-C motif) ligand 7) | 8100971 | 5473 | 3.237 |
| CD226 | CD226 molecule | 8023757 | 10666 | 3.214 |
| REGULATION OF CELL DIFFERENTIATION | | | | |
| GeneSymbol | GeneName | ProbeID | EntrezGeneID | log_2_(FC) |
| ITGB3 | integrin, beta 3 (platelet glycoprotein IIIa, antigen CD61) | 8007931 | 3690 | 4.681 |
| ALOX12 | arachidonate 12-lipoxygenase | 8004221 | 239 | 3.054 |
| GATA1 | GATA binding protein 1 (globin transcription factor 1) | 8167360 | 2623 | 2.918 |
| CA2 | carbonic anhydrase II | 8147132 | 760 | 2.755 |
| PDE5A | phosphodiesterase 5A, cGMP-specific | 8102532 | 8654 | 2.695 |
| TACSTD2 | tumor-associated calcium signal transducer 2 | 7916584 | 4070 | 2.521 |
| CSF1 | colony stimulating factor 1 (macrophage) | 7903786 | 1435 | 2.518 |
| RGCC | regulator of cell cycle | 7968789 | 28984 | 2.518 |
| CAMK1 | calcium/calmodulin-dependent protein kinase I | 8085206 | 8536 | 2.473 |
| PF4 | platelet factor 4 | 8100966 | 5196 | 2.373 |
| EXOCYTOSIS | | | | |
| GeneSymbol | GeneName | ProbeID | EntrezGeneID | log_2_(FC) |
| ITGB3 | integrin, beta 3 (platelet glycoprotein IIIa, antigen CD61) | 8007931 | 3690 | 4.681 |
| ITGA2B | integrin, alpha 2b (platelet glycoprotein IIb of IIb/IIIa complex, antigen CD41) | 8016044 | 3674 | 4.003 |
| SELP | selectin P (granule membrane protein 140kDa, antigen CD62) | 7922200 | 6403 | 3.726 |
| PDGFA | platelet-derived growth factor alpha polypeptide | 8137670 | 5154 | 3.289 |
| PPBP | pro-platelet basic protein (chemokine (C-X-C motif) ligand 7) | 8100971 | 5473 | 3.237 |
| ADORA3 | adenosine A3 receptor | 7918533 | 140 | 3.031 |
| ANK1 | ankyrin 1, erythrocytic | 8150439 | 286 | 2.922 |
| LAT | linker for activation of T cells | 7994541 | 27040 | 2.502 |
| RAB27B | RAB27B, member RAS oncogene family | 8021301 | 5874 | 2.484 |
| CCL5 | chemokine (C-C motif) ligand 5 | 8014316 | 6352 | 2.447 |
| REGULATION OF CELL MOTILITY | | | | |
| GeneSymbol | GeneName | ProbeID | EntrezGeneID | log_2_(FC) |
| ITGB3 | integrin, beta 3 (platelet glycoprotein IIIa, antigen CD61) | 8007931 | 3690 | 4.681 |
| SELP | selectin P (granule membrane protein 140kDa, antigen CD62) | 7922200 | 6403 | 3.726 |
| PDGFA | platelet-derived growth factor alpha polypeptide | 8137670 | 5154 | 3.289 |
| ALOX12 | arachidonate 12-lipoxygenase | 8004221 | 239 | 3.054 |
| ADORA3 | adenosine A3 receptor | 7918533 | 140 | 3.031 |
| TACSTD2 | tumor-associated calcium signal transducer 2 | 7916584 | 4070 | 2.521 |
| CSF1 | colony stimulating factor 1 (macrophage) | 7903786 | 1435 | 2.518 |
| RGCC | regulator of cell cycle | 7968789 | 28984 | 2.518 |
| CCL5 | chemokine (C-C motif) ligand 5 | 8014316 | 6352 | 2.447 |
| NEXN | nexilin (F actin binding protein) | 7902495 | 91624 | 2.086 |
| CELL SURFACE RECEPTOR SIGNALING PATHWAY | | | | |
| GeneSymbol | GeneName | ProbeID | EntrezGeneID | log_2_(FC) |
| ITGB3 | integrin, beta 3 (platelet glycoprotein IIIa, antigen CD61) | 8007931 | 3690 | 4.681 |
| AZU1 | azurocidin 1 | 8024038 | 566 | 4.42 |
| GP1BA | glycoprotein Ib (platelet), alpha polypeptide | 8004024 | 2811 | 4.053 |
| IL5RA | interleukin 5 receptor, alpha | 8085062 | 3568 | 4.031 |
| ITGA2B | integrin, alpha 2b (platelet glycoprotein IIb of IIb/IIIa complex, antigen CD41) | 8016044 | 3674 | 4.003 |
| PDGFA | platelet-derived growth factor alpha polypeptide | 8137670 | 5154 | 3.289 |
| CD226 | CD226 molecule | 8023757 | 10666 | 3.214 |
| GRAP2 | GRB2-related adaptor protein 2 | 8073194 | 9402 | 2.82 |
| GP6 | glycoprotein VI (platelet) | 8039294 | 51206 | 2.781 |
| P2RY2 | purinergic receptor P2Y, G-protein coupled, 2 | 7942400 | 5029 | 2.732 |
| REGULATION OF CELL PROLIFERATION | | | | |
| GeneSymbol | GeneName | ProbeID | EntrezGeneID | log_2_(FC) |
| ELANE | elastase, neutrophil expressed | 8024056 | 1991 | 5.053 |
| ITGB3 | integrin, beta 3 (platelet glycoprotein IIIa, antigen CD61) | 8007931 | 3690 | 4.681 |
| PRTN3 | proteinase 3 | 8024048 | 5657 | 4.142 |
| PDGFA | platelet-derived growth factor alpha polypeptide | 8137670 | 5154 | 3.289 |
| ALOX12 | arachidonate 12-lipoxygenase | 8004221 | 239 | 3.054 |
| GATA1 | GATA binding protein 1 (globin transcription factor 1) | 8167360 | 2623 | 2.918 |
| PDE5A | phosphodiesterase 5A, cGMP-specific | 8102532 | 8654 | 2.695 |
| TACSTD2 | tumor-associated calcium signal transducer 2 | 7916584 | 4070 | 2.521 |
| CSF1 | colony stimulating factor 1 (macrophage) | 7903786 | 1435 | 2.518 |
| RGCC | regulator of cell cycle | 7968789 | 28984 | 2.518 |
